# Zolmitriptan niosomal transdermal patches: combating migraine via epigenetic and endocannabinoid pathways and reversal of migraine hypercoagulability

**DOI:** 10.1007/s13346-024-01731-6

**Published:** 2024-11-05

**Authors:** Nancy Abdel Hamid Abou Youssef, Gihan Salah Labib, Abeer Ahmed Kassem, Nesrine S. El-Mezayen

**Affiliations:** 1 Department of Pharmaceutics and Pharmaceutical technology, Faculty of Pharmacy, Pharos University in Alexandria, Canal El Mahmoudia street, beside Green Plaza Complex , Alexandria, 21648 Egypt; 2 Faculty of Pharmacy, Alamein International University, Alamein, Matrouh 51718 Egypt; 3 Department of Pharmacology and Therapeutics, Faculty of Pharmacy, Pharos University in Alexandria, Alexandria, 21648 Egypt

**Keywords:** Endocannabinoid receptors, Epigenetically-altered genes, Hemostatic pathways, Niosomal patch, Transdermal, Zolmitriptan

## Abstract

Conventional zolmitriptan (ZOL) has limited oral bioavailability, many adverse effects, and poor membrane penetrability that negatively influences its accessibility to its 5-HT_1B/1D_ receptor binding pocket, located transmemberanous. This work aimed at preparing transdermal ZOL-nanoformulation (niosomes) to surpass these limitations and to explore novel antimigraine mechanisms for ZOL via modulation of the epigenetically-altered chronification genes (RAMP-1, NPTX-2) or microRNAs and affecting the endocannabinoid CB-1/MAPK pathway. The prepared ZOL niosomes (F_sp60/6−1:1_) exhibited %EE of 57.28%, PS of 472.3 nm, PDI of 0.366, and ZP of -26 mV were cast into patch with content uniformity of 93.12%, maintained endurance after 200-times folding, no interaction between its components (FT-IR), a biphasic release pattern and good stability after storage at 4 °C for 6 months. In-vivo ZOL-patch application in rats with nitroglycerin-induced migraine showed significant management of migraine pain symptoms and photophobia assessed behaviorally, decreased brain levels of the trigeminal neuronal activation marker (c-fos), the migraine pain neurotransmitter (CGRP) and the serum levels of different migraine pain markers (substance P, nitric-oxide, and TNF-α). It also significantly decreased RAMP-1, NPTX-2, miR-382-5p, and CB-1/MAPK gene expression reflecting improved efficacy and brain receptors delivery to a much greater extent than conventional ZOL has done. Additionally, this nanoformulation significantly opposed migraine-induced platelet activation and hypercoagulable status in both central and peripheral circulations as evidenced by the significant decrease in adenosine diphosphate, thrombin, factor X, CD41, and Von-Willebrand factor levels assessed peripherally and centrally. TPF_sp60/6−1:1_ significantly improved ZOL efficacy and accessibility to brain-receptors to a much greater extent than conventional ZOL-solution.

KeywordsEndocannabinoid receptors; Epigenetically-altered genes; Hemostatic pathways; Niosomal patch; Transdermal; Zolmitriptan

**Graphical abstract:**

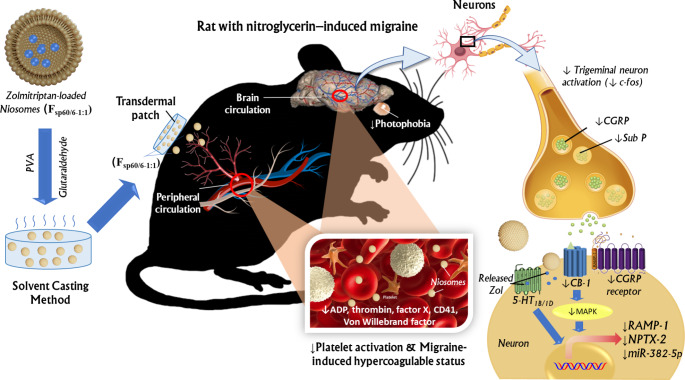

**Supplementary Information:**

The online version contains supplementary material available at 10.1007/s13346-024-01731-6.

## Introduction

Migraine is a primary headache disorder listed as the sixth most disabling symptom globally [1, 2], with a female predominance [3]. The particular migraine pathophysiology is not completely understood. It seems to be a complex genetic predisposition together with behavioral and environmental conditions that alter sensory brain processing with subsequent increased sensory susceptibility [1].

Generally, migraine attacks are characterized by four key phases; the premonitory, the aura, the headache, and the postdrome phases. The headache phase is characterized by the trigeminovascular system activation and nociceptive signals transmission, which causes neurotransmitter release such as calcitonin gene-related peptide (CGRP), and nitric oxide (NO) [4].

Despite advances in therapeutic progress, migraine treatment is still unsatisfying. Triptans, e.g., sumatriptan and zolmitriptan (ZOL) besides nonsteroidal anti-inflammatory drugs are the chief options for acute migraine treatments. Other options for migraine management include gepants and ditans for acute migraine treatment and the novel CGRP inhibitors and receptor blockers as migraine preventive treatment. Beta-blockers, tricyclic antidepressants, and anticonvulsants are also used as preventive therapies for migraine [5]. These myriad drug classes with diverse mechanisms of action intensify the poor understanding of migraine pathophysiology.

Recently, epigenetics provided new insight into migraine pathogenesis and therapeutic response elucidation. Epigenome alteration may lead to the progression to chronic migraine (chronification of migraine), such as the altered methylation pattern of the receptor activity-modifying protein-1 (RAMP-1), and the neuronal pentraxin-2 (NPTX-2) genes. RAMP-1 is a subunit of CGRP that alters the pharmacology, and cell sensitivity to CGRP peptides. The NPTX-2 gene (encodes the NPTX-2 protein) is an inhibitor of excitatory synapses that plays an important role in synaptic plasticity regulation [4]. In addition, epigenetic alterations in the endocannabinoid system (ES) were also reported in migraineurs [4]. The ES’s elements exert antinociceptive effects by activating cannabinoid receptors, especially the cannabinoid type-1 (CB-1) [6]. CB-1 is a G-protein coupled receptor (GPCR) whose primary action involves neuronal transmission. CB-1 signaling is mainly coupled to the mitogen-activated protein kinase (MAPK) pathway that regulates various biological processes and functions by phosphorylation of specific targets (such as transcription factors) [7]. Moreover, CB-1 activation may induce epigenetic alterations; it has been reported to promote changes in the expression of genes controlling various neurotransmitter systems [8].

The field of microRNA-dependent expression regulation and its influence on migraine physiology and pathology is one of the most pertinent areas of epigenetics. Migraine pathology is mainly based on the dysfunction of neurotransmitter systems, receptors, and signaling transductions, which are implicit to be controlled by microRNAs. For instance, evidence pointed to the putative potential of miR-382-5p as a possible diagnostic/prognostic marker reflecting migraine severity and therapeutic response [9]. Based on the initial evidence of the potential role of epigenetics in migraine, it can be inferred that therapeutics modulating the above-mentioned epigenetically-altered genes and miRNA may be valuable therapeutic options for migraine treatment.

Zolmitriptan, a second-generation triptan is a potent selective 5-hydroxytryptamine (serotonin) inhibitory receptor (5-HT_1B/1D_) partial agonist for acute migraine treatment. It inhibits trigeminovascular activation peripherally; produces cranial vasculature constriction and inhibits the extravasation of plasma protein into the dura during trigeminal system stimulation, hence enabling drug access to the trigeminal nucleus caudalis 5-HT_1B/1D_ receptors (not accessible to other triptans) [10, 11]. In addition, it restrains the release of vasoactive neuropeptides. The 5-HT_1B/1D_ receptor ZOL binding pocket is located transmemberanous and thus membrane penetrability is crucial for ZOL accessibility to its binding site. ZOL is categorized as a class III biopharmaceutics classification system (BCS) medication, that shows high solubility and low permeability. Owing to its short half-life of 2.5 h and poor oral bioavailability of about 40% due to hepatic first-pass metabolism and its susceptibility to efflux by P-gp transporters, multiple oral dosing is demanded to resist the recurrence of a migraine attack that needs both fast onset of action and long duration [10, 12, 13]. However, frequent oral administration would increase its associated side effects, including nausea, and vomiting that may cause erratic absorption, dizziness [10], and coronary vasoconstriction, paresthesia, flushing, tingling, neck pain, and chest tightness [14]. Other more hazardous adverse effects of triptans are cardiovascular ones like arrhythmias, myocardial infarctions, and strokes [15]. Further, ischemia-hypoxia causes tenacious deficits in the cerebral perfusion which may lead to platelet deposition and fibrin accumulation within the cerebral circulation [16]. To combat these problems, a substitute non-oral, non-invasive route for the administration of ZOL, was necessary. The transdermal route offers plenty of merits, including, complete avoidance of hepatic first-pass metabolism, and provision of an extended period of administration, that ensures a constant plasma concentration, and consequently, multiple dosing will not be required, and possible side effects will be minimized significantly. Moreover, painless and easy transdermal application will improve patient compliance [12, 17]. Nevertheless, the skin barrier hinders the transdermal permeation of drug molecules. To circumvent this limitation, many chemical and physical approaches were explored, such as using chemical penetration enhancers, microneedles, iontophoresis, nanoformulations, and others [18, 19]. Chemical penetration enhancers, microneedles and iontophoresis are effective tools for transdermal permeation, but each suffers from some problems [20, 21]. It is worth mentioning that a Food and Drug Administration (FDA) approved transdermal sumatriptan iontophoretic transdermal Zecuity^®^ patch for migraine treatment was withdrawn from the market due to FDA safety concerns reported as burns and scarring [22]. Nanocarriers offer an interesting strategy for transdermal delivery, as they can cross the stratum corneum in a non-invasive manner, with low skin irritancy [23]. As mentioned above, ZOL membrane penetrability is critical for better accessibility to its binding site, because the binding pocket of ZOL at the 5-HT_1B/1D_ receptor is located trans-membranous [24]. The minute dimensions and large surface area of nanosized formulations, permit them to penetrate cells [25]. Many literatures investigated nanoformulations of ZOL for different routes of administration: oral [26], oromucosal [27], nasal [28–31], but few had studied this drug for transdermal delivery [32, 33].

Niosomes are one of the most stable vesicular nanocarriers that constitute compatible, and safe nonionic surfactants. Their unique structure enables them to co-deliver both hydrophilic and lipophilic drugs [34]. They are sustainable, simply prepared by self-assembly technique and are easy to scale up in a cost-effective way [35]. Superior merits of niosomes for transdermal application include least irritancy, enhanced drug penetration, and local depot for sustained drug delivery [36]. This perfectly suits for safety and maximum efficacy of the ZOL delivery system, as both fast onset and long duration are crucial parameters to resist the recurrence of a migraine attack. Another advantage in using niosomes is that they don’t cause blood coagulation and additionally they do not generate oxygen-free radicles whatever is the electric charge of them [37]. However, the liquid nature of niosomes prevents proper skin contact at the application site. Consequently, the incorporation of niosomes into gels or patches will guarantee proper residency on the skin.

The specific goals of this research were to develop an optimized niosomal patch formulation for improved transdermal delivery of ZOL and to explore possible novel mechanisms for the antimigraine action of ZOL via alternative pathways, including those affecting the altered migraine chronification genes (RAMP-1, and NPTX-2*)*, or microRNAs, and modulation of the endocannabinoid; CB-1/MAPK pathway. Based on our knowledge, these probable mechanisms were not previously investigated. Furthermore, the influence of such nanoformulation on the migraine-induced hypercoagulable status was investigated.

## Materials and methods

### Materials

Zolmitriptan (ZOL) was supplied from Pharmaceutical Industries, EIPICO, Cairo, Egypt. Cholesterol, Span 60, and Brij 35 were purchased from Sigma Aldrich Inc. (St. Louis, MO). Glutaraldehyde (GA) was purchased from El Nasr pharmaceutical chemicals company, Alexandria, Egypt. Polyvinyl alcohol (PVA), Mowiol^®^ 40–80, was purchased from E.I. du-Pont de Nemours Co (Wilmington, DE, USA). Nitroglycerin (NTG) was supplied from October Pharma (Cairo, Egypt); Isoflurane was supplied from PHARCO pharmaceuticals (Alexandria, Egypt); thrombin activity, adenosine diphosphate (ADP), and nitric oxide (NO) assay kits were purchased from Abcam (Cambridge, United Kingdom); Rat calcitonin gene-related peptide (CGRP), Factor X, tumor necrosis factor-alpha (TNF-α) and substance P ELISA Kits were purchased from CUSABIO (Houston, United States); Rat c-fos and CD 41 ELISA kits were purchased from Assay Genie (Dublin 2, Ireland); Von Willebrand factor ELISA kit was purchased from Elabscience (Texas, United States); High capacity cDNA reverse transcription kit (applied biosystems), Maxima SYBR Green qPCR master mix (Thermo Scientific), and TRIzol plus RNA purification kit (Invitrogen) were all brands under the Life Technologies brand of - ThermoFisher (Carlsbad, United States); RT-PCR reagents kits and universal primers were supplied from Qiagen (Hilden, Germany). All other chemicals or solvents were of analytical or pharmaceutical grades.

### Preparation of niosomal suspension

Niosomal formulations were prepared by the conventional thin film hydration method as was been previously reported [33, 38], with slight change using the surface active agents (SAA) Span 60, and Brij 35 at different hydrophilic-lipophilic balance (HLB) values; (4.7, 6, 8) (Table 1). Adequate proportions of surfactants, used for the required HLB value of a system, were calculated according to the following equation (Eq. 1):

1 $$\:HLB\:mixture=X\:{HLB}_{A}+\left(1-X\right){\:HLB}_{B}$$

Where X is the fraction of SAA having an HLB. A and B are the SAA constituting the system [39].

The calculated amount of either Span 60 and/or Brij 35 and cholesterol was entirely dissolved in 10 mL chloroform and poured into a rounded volumetric flask. Evaporation of chloroform was performed at 60 °C under vacuum, using a rotary evaporator (Rotary Evaporator, Type 110, Buchi, Switzerland) at 135 rpm, so that a delicate dry film was obtained on the inner wall of the flask. Film hydration was done by adding 10 mL of phosphate buffer saline (PBS, pH 7.4), containing ZOL (2 mg/mL) at 60 °C for two hours at 100 rpm. The resultant niosomal suspension was stirred using a magnetic stirrer at 100 rpm for 40 min at 40 °C for maturation, followed by sonication for three minutes (Sonica^®^, Ultrasonic cleaners 2200EP S3, Soltec^®^, Italy). The niosomal suspension was kept at 2–8 °C.

**Table 1:** Composition and characterization of ZOL niosomal formulations

Formula code	HLB	Composition			%EE	Particle size (nm)	PDI
		Span 60	Brij 35	SAA: cholesterol			
F_sp60/4.7-1:1_	4.7	100%	-	1:1	49.72 ± 0.443	NA	
F_sp60/6-1:1_	6	90%	10%	1:1	57.28 ± 2.5	472.3 ± 7.62	0.366
F_sp60/8-1:1_	8	73%	27%	1:1	32.03 ± 0.135	NA	
F_sp60/6-1:0.5_	6	90%	10%	1:0.5	49.11 ± 2.9	448.67 ± 16.57	0.686
F_sp60/6-1:1.5_	6	90%	10%	1:1.5	31.42 ± 0.63	814.47 ± 23.37	0.63

### Characterization of ZOL niosomal formulations

#### Entrapment efficiency

Entrapment efficiency (%EE) of ZOL in different niosomes’ formulations was determined indirectly by ultracentrifugation method using centrisart tubes (Sartorius, 13239E, Germany), with a filter membrane of M.Wt. cut off 10 kDa [40, 41]. Niosomal samples were centrifuged in a cooling centrifuge (SMIC 80 − 2 Centrifuge, China) at 4 °C at 10,000 rpm for 20 min as a preliminary step to obtain a clearer layer. A fixed volume (2 mL) of the separated clear layer was placed in the outer chamber, and the sample recovery chamber was placed on top of the sample and centrifuged (Smic-80-2, China) at 4000 rpm for 15 min The obtained layer was analyzed for unentrapped ZOL using UV spectrophotometer (Shimadzu UV-1800, Shimadzu, Tokyo, Japan) at the predetermined λ _max_ of 283 nm after appropriate dilution, against that of *placebo* niosomes. Each sample was analyzed in triplicate. The %EE was calculated using the following equation (Eq. 2):

2 $$ \% {\text{EE}} = \frac{\left( \begin{array}{l} {\text{Concentration of total}} \\ \quad {\text{ZOL in niosomes}} \end{array} \right) - \left( \begin{array} {l}{\text{Concentration of}} \\ \quad {\text{unentrapped ZOL}} \end{array} \right) } {{\text{Concentration of total ZOL in niosomes}}} \times 100 $$

#### Particle size analysis

Particle size analysis of the prepared niosomes was performed using dynamic light scattering technique (Zetasizer Ver. 7.13, Malvern, Panalytical Ltd, USA). After appropriate dilution with deionized water, particle size (PS), polydispersity index (PDI), and Zeta potential (ZP) were assessed.

#### Morphological characterization of the optimized formula using transmission electron microscope

Transmission electron microscopy (TEM) was used to examine the surface morphology of the optimized niosomal formula. Few drops of diluted F_sp60/6−1:1_ were loaded onto a carbon coated copper grid, stained using 2% uranyl acetate, then dried in air at room temperature and finally tested under TEM (Jeol, JEM-100 CX, Japan).

#### In-vitro release testing

Release study was conducted as previously reported with some modifications [33]. Shaking water bath (Dihan Scientific, Korea) was utilized for this experiment. A glass tube opened from its two ends with 3 cm diameter and 6 cm length was used. A cellophane membrane with a molecular weight cut-off of 12,000–14,000 (VISKING1, Serva Electrophoresis, GmbH, Germany) was presoaked in PBS (pH 7.4) and was securely tied at one end of the tube using a rubber strip. One milliliter of niosomal formulation containing 2 mg ZOL was placed onto the cellophane membrane, inside the glass tube. The surface of the cellophane membrane at the end where the membrane is tied, was adjusted to just touch the surface of the release medium (12 mL of PBS) into a beaker (50 mL volume). The tube was firmly fitted to the beaker by a cork ring and the beaker was fixed to the shaking water bath. The beaker was allowed to shake at 50 stroke/min at 35^°^C ± 0.5^°^C. Two-milliliter samples were removed at regular intervals (0.25, 0.5, 1, 3, 4, 5, 24, and 48 h) for analysis spectrophotometrically at 283 nm and replenished by fresh prewarmed release medium to preserve sink conditions. *Placebo* formulations were used as blank, and ZOL concentration was calculated by reference to the appropriate calibration curve constructed in the same medium. Each experiment was performed in triplicate (*n* = 3 ± SD). The in-vitro release data was analyzed using DD solver software and fitted to four different kinetic models to describe the release kinetics; zero order, first order, Higuchi, and Korsmeyer-Peppas. The model with the highest correlation coefficient was considered to be the best-fitting one.

#### Fabrication of ZOL transdermal niosomal patch (TPFsp60/6−1:1)

*Placebo* patches were prepared by solvent casting method as published before with minor alterations [42]. The calculated amount of PVA was dissolved in hot distilled water at 100 °C with continuous stirring to obtain 4% w/v solution. After complete solubility, the PVA solution was cooled to room temperature and glycerol (7% w/w) as a plasticizer was added. Aqueous GA solution (50%) was mixed with the desired GA: PVA ratio of 1:100 w/w. Four milliliters of the resultant crosslinked polymeric solution with pH 6.2 was poured into glass cups with circular base of 2 cm diameter and dried at 40 °C, for 24 h in an incubator, so that flat and dense patches were obtained.

The optimized niosomal formulation F_sp60/6−1:1_ was freeze-dried using 1% mannitol as a cryoprotectant [43], to obtain a more stable dry form, proniosomes. Samples were frozen at − 80 °C for 24 h in an ultra-low temperature refrigerator. The frozen samples were dried in a freeze-dryer (ilShinBioBase freeze dryer, FDI-0650, Korea) for 48 h, after which the lyophilized niosomes were ready for patch preparation. Appropriate weight of the lyophilized niosomes equivalent to 2 mg ZOL was dispersed homogeneously in 4 mL cool clear cross-linked PVA solution, as previously mentioned, using magnetic stirring, then sonicated for 15–20 min to remove air bubbles. Finally, the mixture was poured in the assigned 2 cm width cup and left to dry at 40 °C, for 24 h in an incubator. Zolmitriptan transdermal patches (TPF_sp60/6−1:1_) were kept at 4 °C for further investigations.

### Characterization of niosomal ZOL transdermal patch

#### Fourier transform infrared spectroscopic analysis (FT-IR)

Pure ZOL, ZOL transdermal patch formulation (TPF_sp60/6-1:1_), and physical mixture were examined by FT-IR (FT-IR, Cary 630, Agilent technology, Malaysia) from 700 to 4000 nm. The physical mixture was prepared by simple mixing of the components with the same ratio in the formulation. The FT-IR spectra were normalized, and the main vibration bands were matched with chemical groups.

#### Physical appearance

The patch was visually inspected for clarity, smoothness, and air bubbles.

#### Drug content uniformity

The drug in one transdermal patch was allowed to dissolve in 12 mL in PBS (pH 7.4) by sonication for 30 min, centrifuged at 14,000 rpm for 20 min, and finally filtered through a 0.45 μm micropore filter. The clear layer was appropriately diluted and measured spectrophotometrically at 283 nm [44]. The same procedure was performed for *placebo* patches and used as a blank. The experiment was repeated five times.

#### Folding endurance

Folding endurance was estimated by repetitive folding of the transdermal patch (TPF_sp60/6-1:1_) at the same location [45]. The folding endurance was considered as the patch was folded 200 times without breaking [46]. The experiments were performed in triplicate.

#### In-vitro release testing

This was performed as previously described for niosomal suspension, where the transdermal patch (TPF_sp60/6−1:1_) was tested instead of the niosomal sample.

#### Stability studies

The optimized ZOL lyophilized niosomes and transdermal patch (TPF_sp60/6−1:1_) were stored in the refrigerator at 4 °C for 6 months. At the end of the storage period, proniosomes were assessed for their %EE, particle size, zeta potential, and morphology. Whereas niosomal ZOL transdermal patch (TPF_sp60/6−1:1_), was evaluated for content uniformity, and folding endurance. Percentage change in each parameter was measured, whenever applicable.

### In-vivo studies

#### Animals

Twenty-eight male locally-bred Sprague-Dawley rats, weighing 200 ± 10 g were purchased from and housed in the animal house of the Faculty of Pharmacy, Pharos University in Alexandria (PUA), Egypt, and were kept under constant environmental conditions during the experimentation period. The local Ethics Committee, PUA, Egypt, approved the use of animals and experimental procedures in the fulfillment of the NIH guidelines for the care and use of laboratory animals (Protocol No: PUA0120233263070) [47].

#### Experimental grouping

Migraine was induced in 21 rats by receiving intraperitoneal (i.p.) NTG (5 mg/kg every other day) for a total of ten days period (5 total injections/rat) [26]. Rats with induced migraine were equally divided into three groups (seven rats/group); the positive control group, represented the untreated rats with NTG-induced migraine, and the two treatment groups. Treatments were administered half an hour after each NTG injection. One treatment group received the standard of care for migraine treatment; conventional ZOL solution (0.45 mg/kg) orally using an oral gavage syringe (This dose is equivalent to a human dose of 5 mg/kg according to Paget’s table) [26]. Zolmitriptan transdermal patches (TPF_sp60/6−1:1_) were applied on the shaved back skin of the rats as the second treatment group. The later rats were housed in one rat/cage to protect the administered patches. Each patch was kept for 48 h until the administration of the next patch, half an hour post NTG injection. These three groups were compared to seven rats that served as a negative control group where rats received five i.p. injections of one mL of saline every other day during the experimentation period (eleven days).

### Behavioral tests

#### Pain assessment

Migraine headache pain was assessed by recording the frequency of cage climbing and head-scratching for 5 min /rat. Since it was reported that NTG-induced pain is obvious after 15 min post NTG injection and lasts for about two hours, migraine pain was assessed 30 min post NTG administration in the positive control group, 30 min after treatment administration in the treatment groups, and twenty-four hours after the last treatment dose administration [48].

#### Photophobia testing

A light/dark box, with dimensions (25 × 30 × 30 cm for each chamber), was used to quantify migraine-induced photophobia criteria and reduced activity. On the test day, each rat was placed into the apparatus for a one-minute acclimation period after which each rat was allowed free access to the entire apparatus. Time spent in the light chamber during a 15-minute test session was recorded [49].

#### Blood and tissue sampling

Twenty-four hours after the last treatment dose, rats were anesthetized using isoflurane anesthesia. Blood samples were collected retro-orbitally and sera were separated. After that, rats were euthanized by decapitation, brains were quickly isolated, washed with ice-cold saline, cut into pieces, and stored at -80 °C until performing biochemical testing, quantitative real time-PCR (qRT-PCR), and ELISA techniques.

### Biological tests

#### Biochemical determination of thrombin activity

For thrombin activity determination, brain samples were extracted with 0.1 M PBS (pH 7.4) containing 1% triton X-100, centrifuged at 14,000 X g for 20 min, and supernatants were collected. Thrombin activity was determined in the prepared supernatants of brain tissue (U/mg protein) and serum samples (U/mL) using proper dilution biochemically according to the manufacturer’s instructions (Cat. #: ab234620) [50].

#### Biochemical determination of brain ADP

For ADP determination in brain tissue (mmol/g tissue), brain samples were homogenized in the provided assay buffer with a homogenizer sitting on ice (10–15 passes). Samples were then centrifuged using a cooling-centrifuge at 15,000 rpm at 5 °C for 15 min. ADP was assessed in the collected supernatants according to the protocol provided with the used kit (Cat. #: ab83359) [51].

#### Biochemical determination of serum nitric oxide (NO)

Nitric oxide (µM/L) assessment was done directly on the isolated serum samples according to the steps provided by the kit’s supplier (Cat. #: ab65328) [52].

#### Determination of brain c-Fos, CGRP, CD 41, and Von Willebrand factor by ELISA

Within brain tissue, c-Fos (ng/mg protein), CGRP (pg/mg protein), CD 41 (ng/mg protein), and Von Willebrand Factor (ng/mg protein) were measured by ELISA kit according to their manufacturers’ manuals (Cat. #: RTEB0846, CSB-E08211r, RTFI00001, and E-EL-R1079, respectively) [53–56].

Brain tissues were homogenized at 15,000 rpm at 5 °C for 5 min in PBS (pH 7.4) to get 20% homogenates using a cooling-centrifuge. c-Fos, CGRP, CD 41, and Von Willebrand Factor were tested in the supernatants obtained following centrifugation [57, 58].

#### Determination of serum substance P, TNF-α, factor X, and CD 41 by ELISA

The levels of substance P (pg/mL), TNF-α (pg/mL), Factor X (ng/mL), and CD 41 (ng/mL) were determined in collected sera using their specific rat ELISA kits according to instructions as stated by their suppliers (Cat. #: CSB-E08358r, CSB-E11987r, CSB-E08441r, and RTFI00001, respectively) [55, 59–62].

#### Assessment of NPTX-2, RAMP-1, CB-1, MAPK-1, and Mir 382-5p content in brain tissues by qRT-PCR

The Total RNA was purified using the TRIzole plus RNA purification kit according to the instructions provided by the kit’s manufacturer. The isolated RNA concentration and purity were spectrophotometrically determined by measuring absorbances at 260 and 280 nm, where the A260/A280 ratio of 1.8-2.0 corresponds to 90–100% purity, respectively. This was followed by reverse transcription utilizing the high-capacity cDNA reverse transcription kit, then amplification was done by thoroughly mixing the reaction mixture (25 µL) according to the manufacturer’s instructions. The reaction mixture comprised Rotor-Gene RT mix (0.25 µL), template RNA (1 µL), RNase-free water (6.5 µL), and Rotor-Gene SYBR Green RT-PCR master mix (12.5 µL). Gene-specific primer sets (5 µL) (shown in Table 2) were used for cDNA amplification. During the extension step, collection of data acquisition was done using Rotor-Gene Q-Pure detection, software version 2.1.0 (build 9); Qiagen, Hilden, Germany. Data was expressed as a normalized ratio of the values of the threshold cycles (Ct) of target genes relative to the reference gene relative expression in the same sample using the ΔΔCt method [63, 64].

**Table 2:** Oligonucleotide primers used for qRT-PCR

Primer	Sequence		Accession number
**NPTX-2**	Forward	5’- GGCATCTATTCCCGAGTTCA − 3’	NM_001034199.1
	Reverse	5’- CCACCAAAGAACAAAGCAGT − 3’	
**RAMP-1**	Forward	5’- AGATGAACCTCATGTCTGCAT − 3’	XM_039084148.1
	Reverse	5’- CAATCTTGTTTGCCACGAGT-3’	
**CB-1**	Forward	5’- TCTGCAGGAAGCCTTTCATC − 3’	XM_017593151.2
	Reverse	5’- TTAGGCCAGACTCAAGGTGA-3’	
**MAPK-1**	Forward	5’- GCTTTGTTCATCCTGCAGTT-3’	XM_006248659.4
	Reverse	5’- TCTGCGGTTGTGCTTTATTC-3’	
β-actin*	Forward	5’- ATGTGGCTGAGGACTTTGATT-3’	XM_039089807.1
	Reverse	5’- ATCTATGCCGTGGATACTTGG-3’	
**Mir 382-5p**	Purchased from Qiagen (Gene Globe ID: YP00204169)		
U6 snRNA*	Purchased from Qiagen (Gene Globe ID: YP02119464)		

* Reference genes.

### Statistical analysis

Using IBM SPSS software package version 20.0 (Armonk, NY: IBM Corp), data from the current study was fed and analyzed. The normality of the distribution was verified using the Kolmogorov-Smirnov test. The mean and standard deviation (SD) were used to describe quantitative data. The analysis of variance (ANOVA; F test) was used for normally distributed quantitative data comparisons among the different groups and was followed by a Post Hoc test (Tukey) for pairwise comparison. The 5% level was used to judge the significance of the obtained results.

## Results

In our study, ZOL niosomal formulations were prepared using cholesterol with the non-ionic SAA Span 60 and Brij 35. Different HLB values of SAA were tested seeking maximum entrapment efficiency for ZOL. Then different cholesterol ratios were examined for the selected formula. Table 1 shows the composition and characterization parameters of ZOL niosomal formulations. The selected formula F_sp60/6−1:1_ was exploited to fabricate a transdermal niosomal patch containing ZOL for the treatment of migraine. The patch was then evaluated for its physical appearance, drug content, folding endurance, in-vitro release behavior, and stability for 6 months. The patch was finally examined in vivo to investigate possible novel mechanisms for the antimigraine action of ZOL via alternative pathways affecting the altered migraine chronification genes (RAMP-1, and NPTX-2*)*, or microRNAs and modulation of the endocannabinoid; CB-1/MAPK pathway.

### Characterization of ZOL niosomal formulations

### Entrapment efficiency, particle size, and polydispersity index

Preliminary tests were conducted to determine the highest %EE based on the HLB of either Span 60 (HLB = 4.7) and a mixture of Span 60 with Brij 35 (Brij HLB = 16.9) in different ratios. The obtained HLB values ranged from 4.7 to 8 (Table 1). The formula F_sp60/6−1:1_ with HLB 6 exhibited the highest %EE of 57.28% among other formulations with varying amounts of Span 60: Brij 35 and fixed SAA: cholesterol ratio of 1:1. The formulae F_sp60/4.7−1:1_ (HLB 4.7) and F_sp60/8−1:1_ (HLB 8) resulted in %EE of 49.72%, and 32.03%, respectively. The formula F_sp60/6−1:1_ with HLB 6 was further tested for varying ratios of SAA: cholesterol; 1:0.5 and 1:1.5. Their %EE were 49.11 and 31.42%, PS 448.67 and 814.47 nm and PDI 0.686 and 0.63, respectively. Hence the formula F_sp60/6−1:1_ with HLB 6, highest %EE of 57.28%, PS of 472.3 nm and PDI of 0.366, was selected for additional assessment.

### Evaluation for the optimized ZOL niosomal formulation (Fsp60/6 − 1:1)

**Fig. 1:**
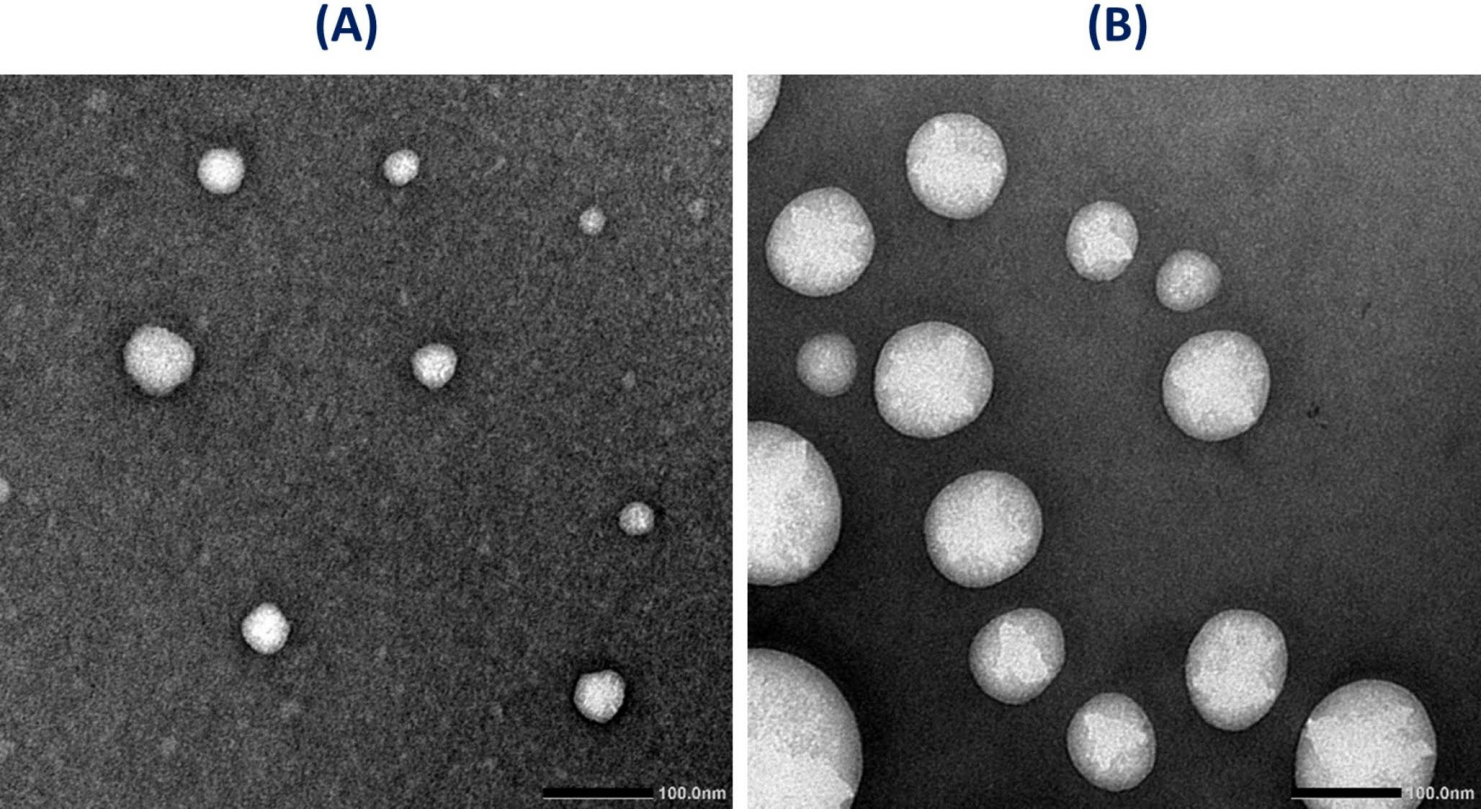
TEM photomicrograph of the fresh optimized ZOL niosomal formulation (F_sp60/6−1:1_) (**A**) and lyophilized (F_sp60/6−1:1_) after storage at 4 °C for 6 months (**B**)

Reasonable stability of the optimized niosomal formulation Fsp60/6-1:1 was confirmed by its zeta potential value of -26 mV. TEM photomicrograph of Fsp60/6-1:1 in Fig. 1A revealed spherical non-aggregating niosomes with a significantly smaller PS than that obtained by the zeta-sizer (Table 1).

### Characterization of ZOL niosomal transdermal patch

#### Fourier transform infrared spectroscopic analysis (FT-IR)

**Fig. 2:**
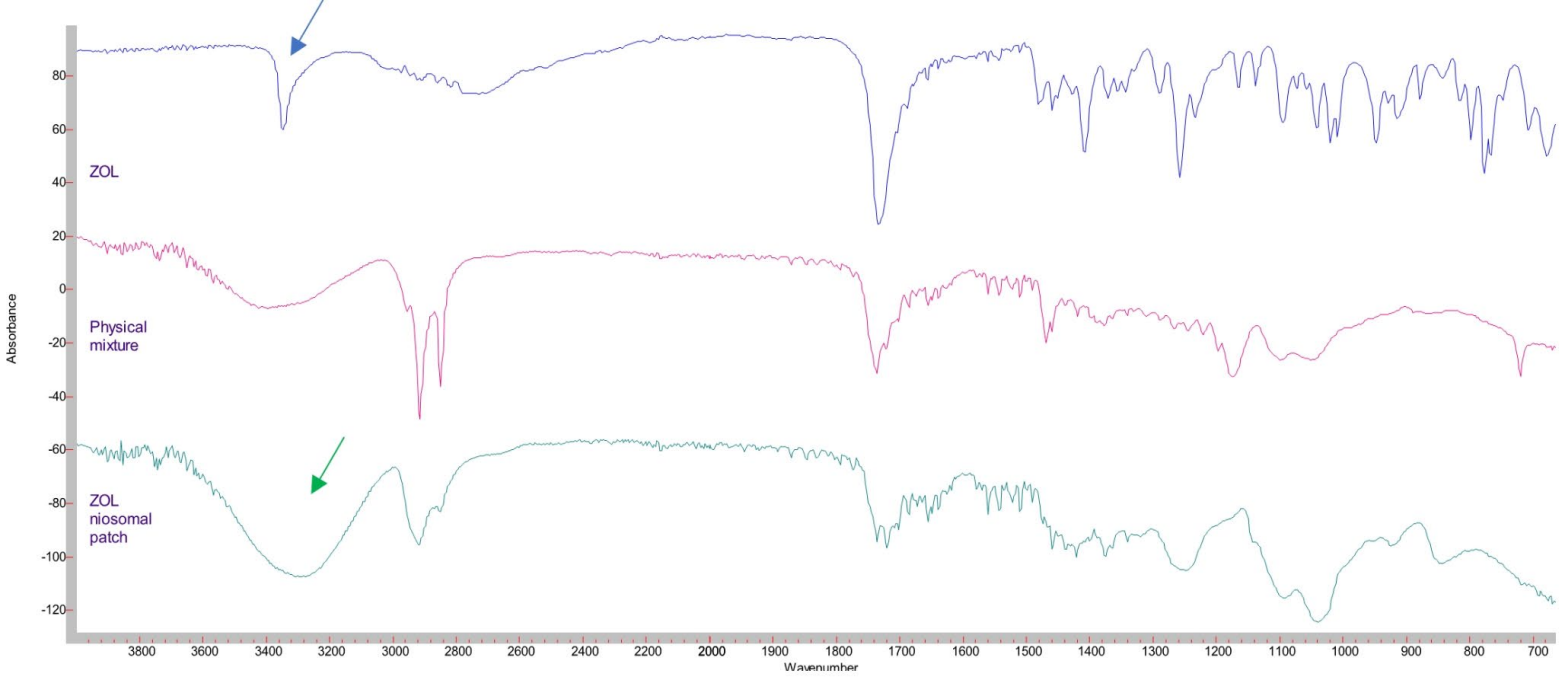
Fourier transform infrared spectroscopic analysis (FT-IR) for: Drug (ZOL), Physical mixture and ZOL transdermal niosomal patch formulation (TPF_sp60/6-1:1_)

A compatibility study by FT-IR for ZOL, physical mixture, and optimized ZOL transdermal patch formulation (TPF_sp60/6−1:1_) is illustrated in Fig. 2. ZOL demonstrated N–H stretching band at 3345.3 cm^− 1^, and C = O stretching band at 1730.3 cm^− 1^ which is comparable to that published earlier [33]. The FT-IR spectrum of transdermal patch formulation exhibited N-H stretching band at 3276.7 cm^− 1^, and C = O stretching at 1733.1 cm^− 1^.

#### Physical appearance, drug content uniformity, and folding endurance

Zolmitriptan niosomal transdermal patch (TPF_sp60/6-1:1_) displayed a smooth texture without air bubbles. The average ZOL content uniformity in five different patches was estimated as 93.12 ± 0.784% of the original amount added (2 mg). For the folding endurance experiment, no breaking happened to any patch after 200 times folding at the same site.

#### In-vitro release study and release kinetics

**Fig. 3:**
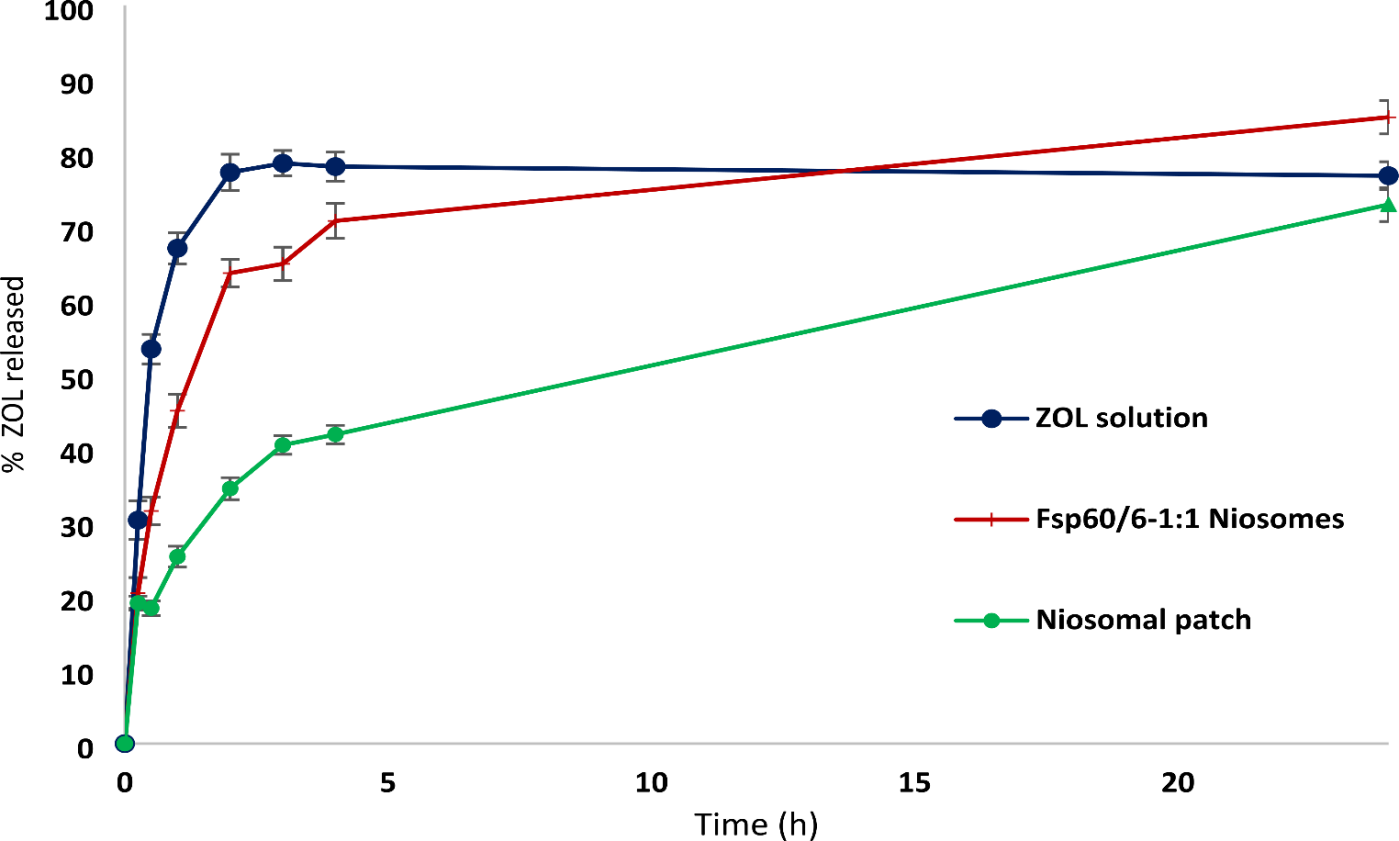
In vitro release profiles of ZOL solution, optimized ZOL niosomal formulation (F_sp60/6−1:1_) and ZOL niosomal transdermal patch (TPF_sp60/6−1:1_)

Figure 3 represents the in vitro release profiles of ZOL solution, optimized ZOL niosomal formulation (F_sp60/6-1:1_), and ZOL niosomal transdermal patch (TPF_sp60/6-1:1_). The release rate of ZOL could be ordered as follows: ZOL solution > niosomes (F_sp60/6-1:1_) > ZOL niosomal transdermal patch (TPF_sp60/6-1:1_). Fast release rate of ZOL from solution and niosomes from 0.5 till 4 h ranged from 30.26 to 78.14% and from 31.5 to 70.79%, respectively. Whereas the niosomal patch showed a slower release rate ranging from 18.34 to 41.87% over the same period. After 48 h niosomal patch showed % drug release of 48.99%.

The release kinetics of zolmitriptan from the ZOL niosomal transdermal patch best fitted to Korsmeyer-Peppas model with the highest R^2^ value of 0.993 and n value of 0.316.

### Stability studies

A stability study was performed for the optimized ZOL lyophilized optimized niosomes (F_sp60/6−1:1_) and ZOL transdermal patch after storage at 4 °C for 6 months. For the niosomes, the decrease in %EE was insignificant (5.78%), and a slight change in PDI (from 0.366 to 0.483) and ZP (from − 26 to -21.3 mV) was recorded. The particle size was increased (Fig. 1A-B) with persistent non-aggregating spherical-shaped niosomes at the end of the study. Concerning the niosomal ZOL patch, no physical change was observed, and the folding endurance was maintained with no breakage after 200 times folding. Only a slight decrease in drug content (5%) was detected.

### Influence of ZOL transdermal patches Fsp60/6 − 1:1 versus oral ZOL solution on migraine headache, serum pain markers, and symptoms assessed biochemically and behaviorally

#### Effect on migraine headache pain

**Fig. 4:**
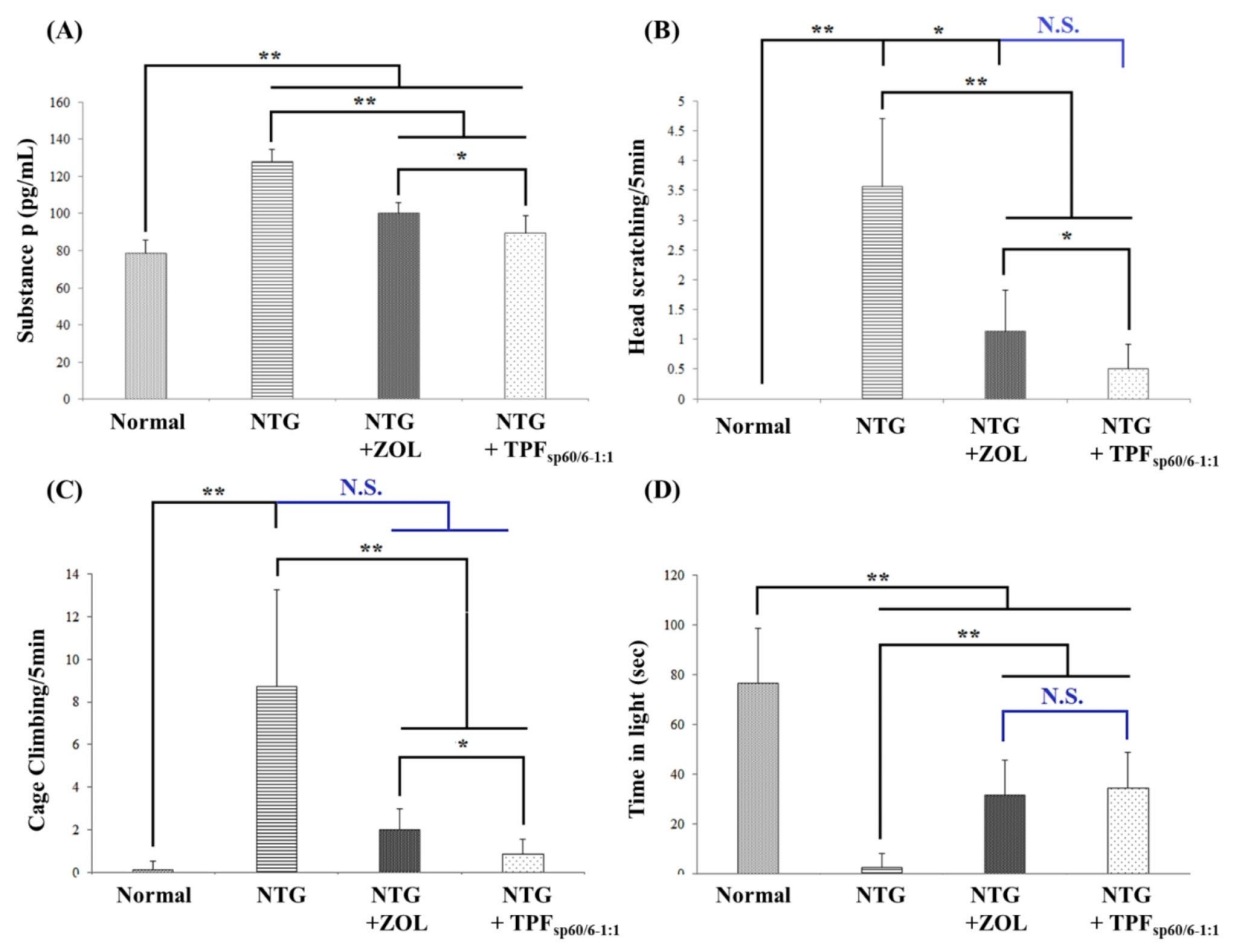
Bar charts for comparing the effect of ZOL transdermal patches (TPF_sp60/6−1:1_) *versus* oral ZOL on migraine headache serum pain markers and symptoms assessed biochemically and behaviorally. **A**: serum levels of substance-P assessed by ELISA; **B**: frequency of head scratching/5 min; **C**: frequency of cage climbing / 5 min; **D**: time spent in light (sec) in the light/dark box for photophobia testing. ANOVA test was used to compare the different groups with Post Hoc Test (Tukey) and Post Hoc Test to compare different receptors for the same group. *: Statistically significant at *p* ≤ 0.05, **: Statistically significant at *p* ≤ 0.001, N.S: Statistically non-significant (*p* > 0.05), *n* = 7; all results are presented as mean ± SD. (NTG: nitroglycerin, ZOL: zolmitriptan, TPF_sp60/6−1:1_: transdermal patches loaded with niosomes)

Migraine pain was assessed biochemically by measuring serum levels of substance P, and behaviorally by recording the number of cage-climbing and head-scratching for 5 min for each rat in treated and untreated groups. The mean serum level of substance P was significantly higher in the group of positive control (NTG-treated) compared to the normal untreated group (*p* < 0.001). Normal rats with no migraine induction showed no signs of pain (no head scratching and minimal cage-climbing). On the other hand, rats with NTG-induced migraine showed a significant rise in the frequency of head scratching and cage climbing indicating pain suffering. Both treatments significantly decreased the level of substance P in serum, and signs of pain compared to the untreated positive control group (*p* < 0.001). There was a statistically significant difference in substance P serum levels between rats receiving oral ZOL and those who received transdermal patches loaded with F_sp60/6−1:1_. The latter group showed significantly less frequent head scratching and cage-climbing (*p* < 0.05), Fig. 4A-C.

#### Effect on migraine photophobia behavior

Herein, induction of migraine with NTG significantly increased light sensitivity and reduced time spent in light compared to negative control rats (*p* < 0.001). Treatment with either TPF_sp60/6−1:1_ or ZOL solution significantly increased time spent in light compared to positive control rats (*p* < 0.001), with a more significant increase in time spent in light in rats treated with TPF_sp60/6−1:1_ (*p* > 0.05), Fig. 4D.

### Influence of ZOL transdermal patches Fsp60/6 − 1:1 versus oral ZOL solution on key migraine pathologic markers

In the current investigation, multiple migraine pathologic criteria markers were assessed; these include NO; a migraine attack inducer and is involved in pain transmission [65] c-fos; a marker of trigeminovascular system activation that relates to cephalic pain [66], CGRP; a neurotransmitter released from the activated trigeminovascular system and is believed to play a causative role in migraine [67], and TNF-α; a proinflammatory molecule involved in migraine neurogenic neuroinflammation and has a key role in migraine transition from episodic to chronic one [68].

**Fig. 5:**
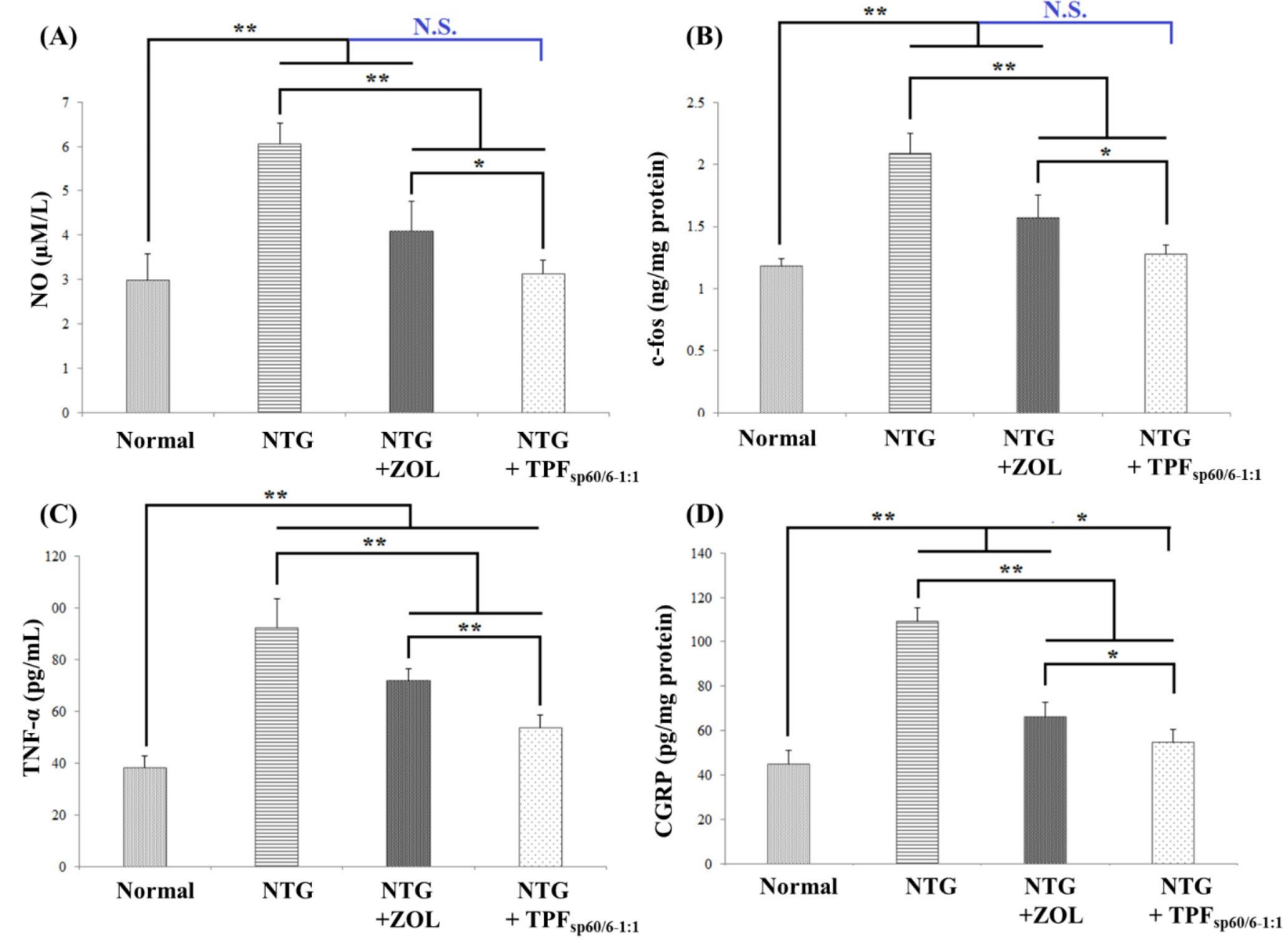
Bar charts for comparing the effect of ZOL transdermal patches (TPF_sp60/6−1:1_) *versus* oral ZOL on key migraine markers. **A**: serum levels of NO; **B**: brain levels of c-fos; **C**: serum levels of TNF-α; **D**: brain levels of CGRP. ANOVA test was used to compare the different groups with Post Hoc Test (Tukey) and Post Hoc Test to compare different receptors for the same group. *: Statistically significant at *p* ≤ 0.05, **: Statistically significant at *p* ≤ 0.001, N.S: Statistically non-significant (*p* > 0.05), *n* = 7; all results are presented as mean ± SD. (NTG: nitroglycerin, ZOL: zolmitriptan, TPFsp60/6 − 1:1: transdermal patches loaded with niosomes, NO: nitric oxide, TNF-α: tumor necrosis factor-alpha, CGRP: calcitonin gene-related peptide)

It was revealed that induction of migraine by NTG caused a significant rise in the mean value of serum NO and c-fos as well as brain tissue levels of CGRP and TNF-α relative to the normal rats group without migraine induction (*p* < 0.001). Treatment with either oral ZOL or transdermal patches loaded with Fsp60/6 − 1:1 significantly decreased the levels of all the assessed migraine pathologic parameters compared to the untreated rats with NTG-induced migraine (*p* < 0.001). The lowest levels were observed in rats receiving transdermal patches loaded with Fsp60/6 − 1:1 and a lack of statistically significant difference between this group and normal rats was detected upon assessing serum levels of NO and c-fos (Fig. 5).

### Influence of ZOL transdermal patches (TPFsp60/6 − 1:1) versus ZOL oral solution on potential novel ZOL mechanisms for treating migraine headache

#### The influence on the epigenetically altered migraine genes and miR-382-5p

**Fig. 6:**
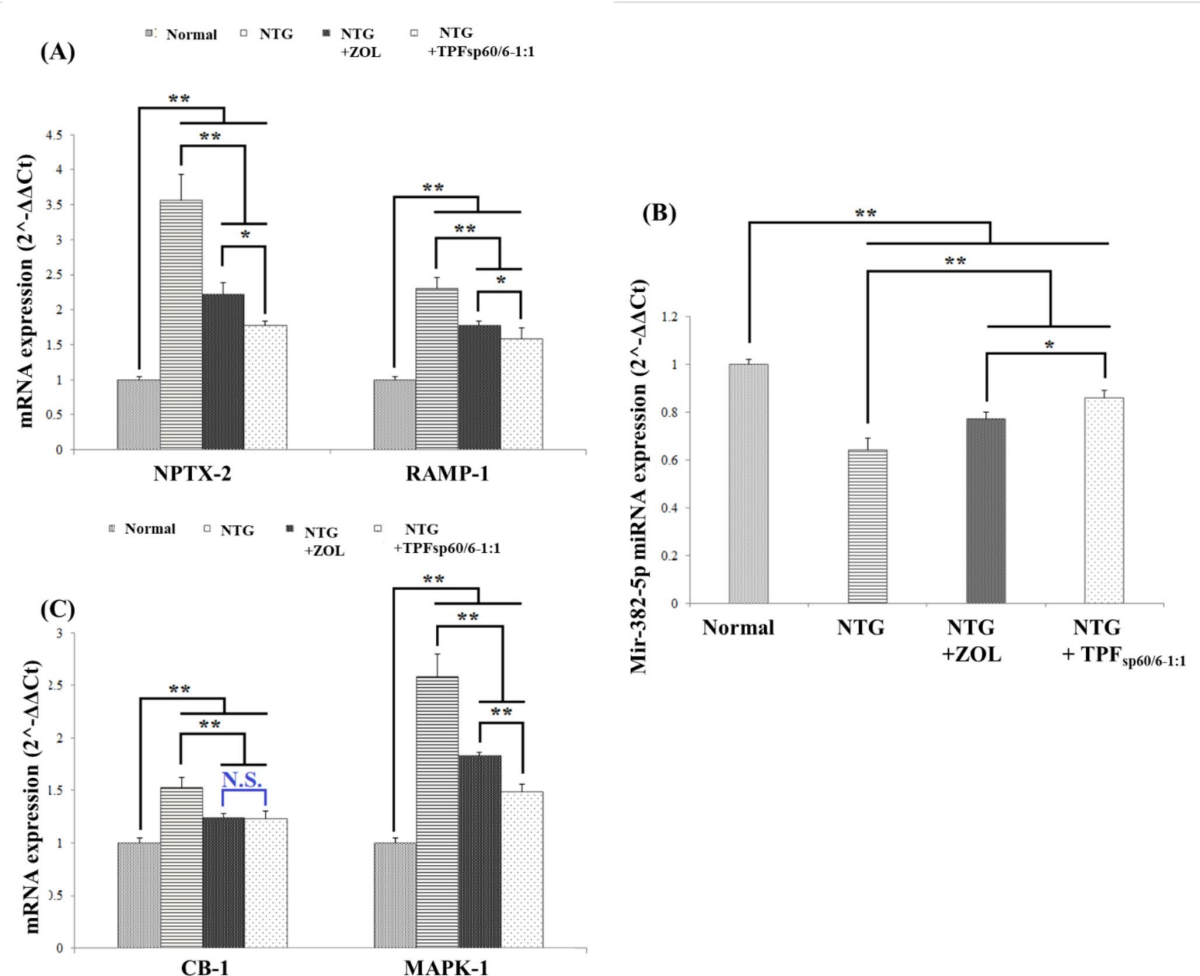
Bar charts for comparing the effect of ZOL transdermal patches (TPF_sp60/6−1:1_) *versus* oral ZOL on potential novel ZOL mechanisms for treating migraine headaches. **A**: gene expression of epigenetically-altered genes in migraine (NPTX-2 and RAMP-1) in brain tissue; **B**: gene expression of Mir-382-5P in brain tissue; **C**: gene expression of cannabis receptor pathway components (CB-1 and MAPK) in brain tissue. ANOVA test was used to compare the different groups with Post Hoc Test (Tukey) and Post Hoc Test to compare different receptors for the same group. *: Statistically significant at *p* ≤ 0.05, **: Statistically significant at *p* ≤ 0.001, N.S: Statistically non-significant (*p* > 0.05), *n* = 7; all results are presented as mean ± SD. (NTG: nitroglycerin, ZOL: zolmitriptan, TPFsp60/6 − 1:1: transdermal patches loaded with niosomes, CB-1: cannabinoid type-1 receptor, MAPK: mitogen-activated protein kinase)

Results revealed a significant increase in the expression of NPTX-2 and RAMP-1 genes and a significant decrease in the miR-382-5p in the brain tissue of rats with NTG-induced migraines relative to normal rats without migraine induction (*p* < 0.001). Treatment with either conventional ZOL or the application of transdermal patches loaded with ZOL niosomes significantly decreased NPTX-2 and RAMP-1 gene expression and increased miR-382-5p expression (*p* < 0.001), with significantly greater efficacy of ZOL transdermal patches (TPF_sp60/6−1:1_) (*p* < 0.05), Fig. 6A, B.

#### The influence on CB-1/MAPK-1 pathway

In the present work, induction of migraine headache with NTG resulted in a significant increase in the gene expression of CB-1 and MAPK-1 in rats’ brain tissues as compared to that of normal rats (*p* < 0.001). ZOL treatment either orally or *via* ZOL transdermal patches TPF_sp60/6−1:1_ significantly corrected the altered CB-1/MAPK-1 pathway. Of note, treatment with ZOL transdermal patches TPF_sp60/6−1:1_ more significantly reduced the level of MAPK-1, but not CB-1, gene expression compared to that of oral ZOL-treated rats (*p* < 0.001), Fig. 6C.

### Influence of ZOL transdermal patches (TPFsp60/6 − 1:1) versus ZOL oral solution on hemostatic pathways in blood and brain tissue

Assessment of a single clotting marker does not accurately reflect the entire physiological process of coagulation. Thus, multiple markers for different stages of the hemostatic pathways were investigated in the blood and brain tissue to reflect the nearly actual influence of tested drugs on hemostasis.

#### Influence on hemostatic pathways in blood

**Fig. 7:**
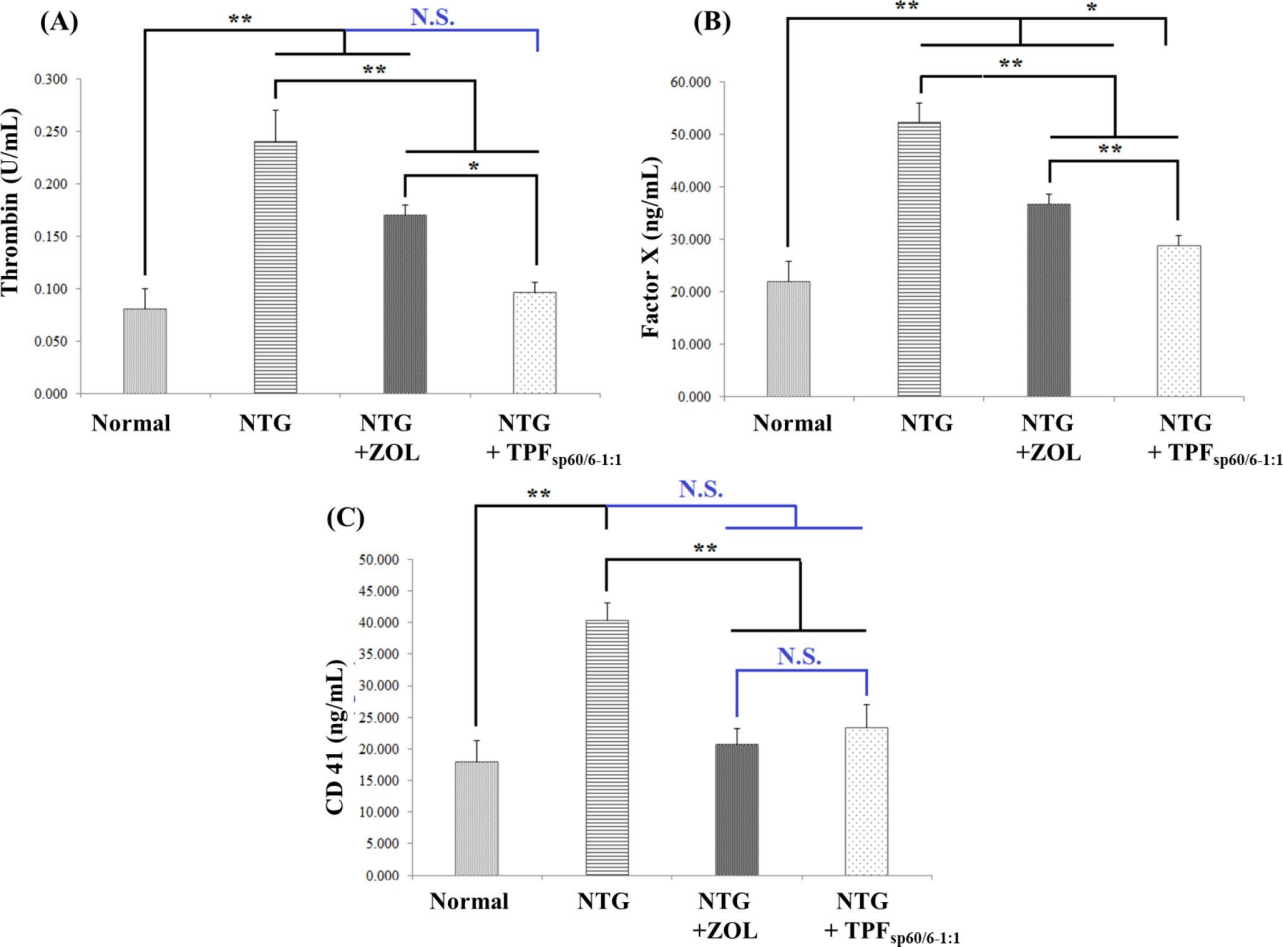
Bar charts for comparing the effect of ZOL transdermal patches (TPF_sp60/6−1:1_) *versus* oral ZOL on hemostatic pathways in blood: **A**: thrombin activity; **B**: factor X level; **C**: CD 41 level. ANOVA test was used to compare the different groups with Post Hoc Test (Tukey) and Post Hoc Test to compare different receptors for the same group. *: Statistically significant at *p* ≤ 0.05, **: Statistically significant at *p* ≤ 0.001, N.S: Statistically non-significant (*p* > 0.05), *n* = 7; all results are presented as mean ± SD. (NTG: nitroglycerin, ZOL: zolmitriptan, TPF_sp60/6−1:1_: transdermal patches loaded with niosomes)

Three markers were assessed in collected sera; the platelet activation marker; CD41, and two clotting factors; thrombin (factor II) and factor X. As compared to normal rats, rats with NTG-induced migraine showed significantly higher mean values of all these markers (*p* < 0.001). ZOL treatments significantly decreased the levels of these markers compared to untreated rats (*p* < 0.001). Notably, except for CD 41, TPF_sp60/6−1:1_ treatment showed significantly lower levels of all these coagulation markers compared to oral ZOL and its influence on thrombin levels was comparable to normal rats, Fig. 7.

#### Influence on hemostatic pathways in brain tissue

**Fig. 8:**
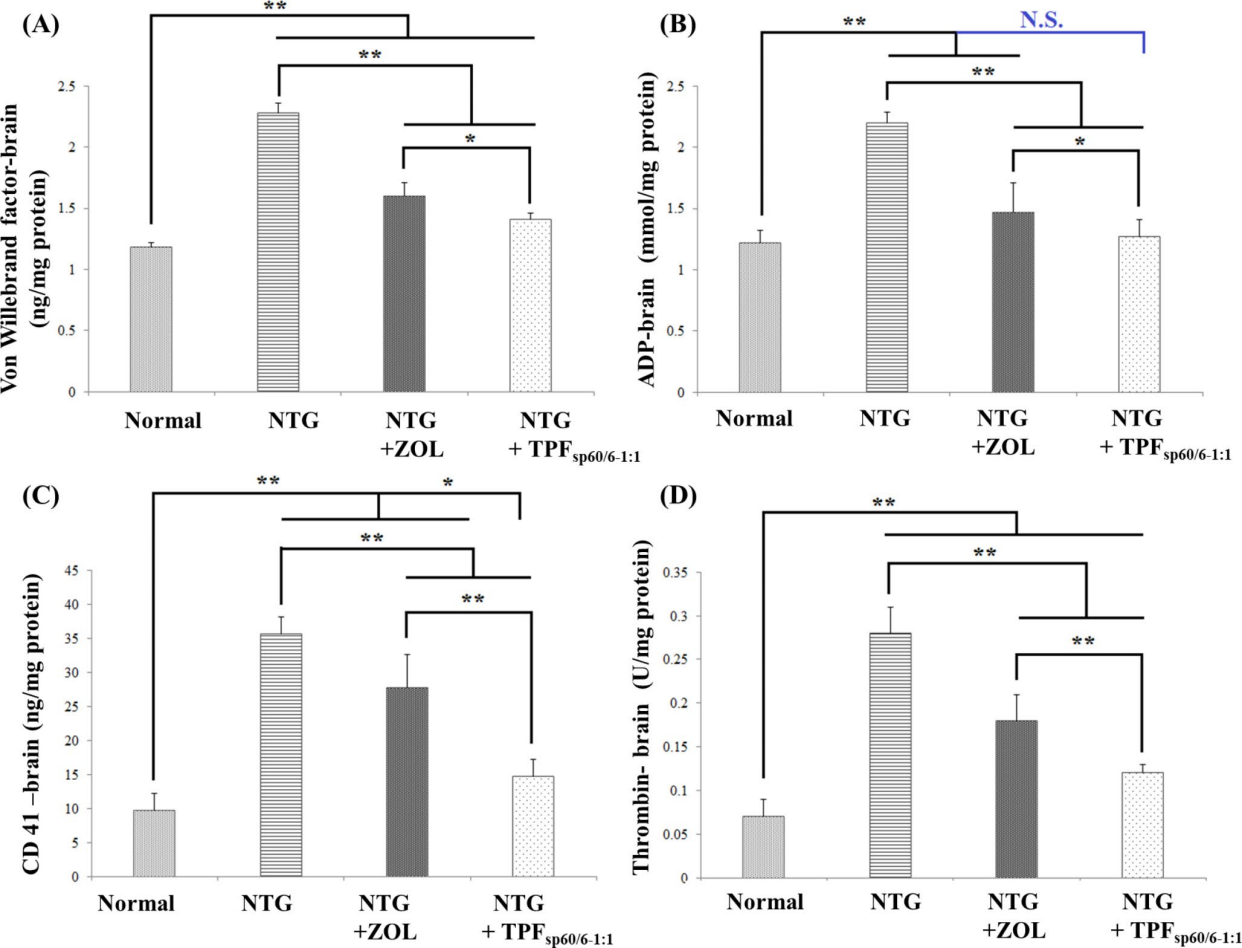
Bar charts for comparing the effect of ZOL transdermal patches (TPF_sp60/6−1:1_) *versus* oral ZOL on hemostatic pathways in brain tissue: **A**: Von Willebrand factor level; **B**: ADP level; **C**: CD 41 level; **D**: thrombin activity. ANOVA test was used to compare the different groups with Post Hoc Test (Tukey) and Post Hoc Test to compare different receptors for the same group. *: Statistically significant at *p* ≤ 0.05, **: Statistically significant at *p* ≤ 0.001, N.S: Statistically non-significant (*p* > 0.05), *n* = 7; all results are presented as mean ± SD. (NTG: nitroglycerin, ZOL: zolmitriptan, TPF_sp60/6−1:1_: transdermal patches loaded with niosomes)

In brain tissue, von Willebrand factor and ADP levels were assessed besides thrombin and CD41. Herein, the induction of migraine using NTG significantly elevated the mean levels of all tested markers compared to normal migraine-free rats (*p* < 0.001). Oral ZOL solution and transdermal TPF_sp60/6−1:1_ significantly decreased the mean levels of all tested coagulation markers compared to untreated positive controls (*p* < 0.001), with clear significant superiority of TPF_sp60/6−1:1_ in decreasing the levels of these markers, Fig. 8.

## Discussion

So far, migraine treatment is still unsatisfying despite the advances in migraine therapeutic progress. Zolmitriptan is one of the triptans that are considered one of the chief options for acute migraine treatments [5]. Conventional ZOL has limited oral bioavailability and many central and cardiovascular adverse effects. In addition, ZOL brings about cerebral ischemia that may induce cerebral coagulation [15, 16]. Further, ZOL has poor membrane penetrability which may negatively influence its accessibility to its binding pocket at the 5-HT_1B/1D_ receptor that is located transmemberanous and thus may result in less efficacy [24]. Therefore, the current study aimed at preparing transdermal ZOL nanoformulation (niosomes) for the purpose of surpassing the limited bioavailability, poor membrane penetrability to better access the ZOL binding site at the 5-HT_1B/1D_ receptor and decreasing the incidence of adverse effects. To avoid leakage at the application site, the niosomal formulation was incorporated into a patch form. Another aim was to investigate, for the first time, the influence of conventional ZOL on epigenetically altered genes, miR-382-5p, and CB-1/MAPK-1 pathway in comparison with nanoformulated ZOL.

As mentioned above, ZOL niosomal formulations were prepared using cholesterol with the non-ionic SAA/s Span 60 and/or Brij 35. It was reported that both the chain size and length of the non-ionic SAA affect the % EE of the drug. Non-ionic SAAs having stearyl (C18) chains exhibit higher %EE compared to those with lauryl (C12) chains [38]. That’s why Span 60 (sorbitan monostearate with long saturated alkyl chain) was chosen as the niosomal formulation SAA. Additionally, the high phase transition temperature and low HLB (4.7) of Span 60 ensure a higher %EE ability compared to other Span family [69, 70]. Different HLB values of SAA were examined to ensure good vesicle formation and to achieve the best % EE for ZOL (Table 1). HLB is an important marker for the niosomal forming capability of a given surfactant. For Span, a HLB number of between 4 and 8 was found to be compatible with niosomal vesicle formation [71]. In addition, HLB plays an important role in monitoring the %EE of the drug inside the niosomal vesicle [70]. The highest %EE was obtained with Nimesulide niosomes having HLB value of 8.6 and it decreased as the HLB value decreased from 8.6 to 1.8 [72]. Thus, we attempted to adjust the HLB in the recommended range by trying combinations of Span 60; HLB value (4.7), and Brij 35 of HLB (16.9) with varying amounts, to obtain HLB values of (4.7, 6, 8) calculated according to the previously mentioned equation, with a total SAA to cholesterol ratio of 1:1. Increasing HLB value of the SAA(s) used from 4.7 to 6, lead to an increase in %EE of ZOL from 49.72 to 57.28%, but further increase in HLB to 8, resulted in a decrease in % EE to 32.03% (Table 1). So the formula (F_sp60/6−1:1_) with the highest %EE was selected for further optimization by testing different SAA: cholesterol ratios: 1:0.5, 1:1 and 1:1.5. Cholesterol plays an important role in niosomal vesicle formation and stability depending on the HLB value. For HLB more than 6, cholesterol is crucial for the formation of the bi-layered vesicle. Whereas for lower HLB values, it improves the stability of the vesicles [70]. Increasing cholesterol ratio in the formula F_sp60/6−1:1_ compared to formula F_sp60/6−1:0.5_, resulted in increased %EE from 49.11 to 57.28%. This could be explained by that cholesterol increases the viscosity of the formulation, improves membrane rigidity, stability, and decreases bilayer permeability [69, 73]. However, more increase in cholesterol ratio in the formula F_sp60/6−1:1.5_ resulted in a decrease in %EE to 31.42% and this could be explained by that an increase in cholesterol ratio above a certain limit may result in disruption of the vesicle structure and hence decreased %EE [74]. The effect of cholesterol concentration was also reflected on vesicular size, as increased cholesterol ratio resulted in increased vesicular size (Table 1). At low cholesterol concentration, the bilayer is assumed to be intimately packed, then when the concentration of cholesterol increases, the hydrophobicity of the membrane increases thus increasing vesicle size [75].

The PS of the optimized formula F_sp60/6−1:1_ under TEM is notably smaller than that obtained by zeta-sizer. This may be due to the technique of nanoparticles’ preparation for TEM imaging which necessitates sample dehydration. While, particle size obtained from zeta-sizer determines the apparent size, which includes the aqueous layer around the particles [76].

The optimized niosomes formulation F_sp60/6-1:1_ not only exhibited the highest entrapment efficiency but also the lowest PDI value of 0.36 among prepared niosomes formulations, indicating the homogeneity of the formulation and a good zeta potential value of -26 mV, confirming reasonable stability.

Results from the compatibility studies by FT-IR concluded that the slight shifting in the band ranges of ZOL transdermal patches (TPF_sp60/6−1:1_), compared to ZOL itself suggested lack of any possible interactions with conservation of main functional groups’ regions of the drug. The probable change in the physical characteristics of the drug in the formulation compared to pure drug may justify the non-superimposed fingerprint region. These results are in accordance with a previously reported study [33].

The rationale behind incorporating the optimized niosomal formulation into a patch was to ensure ease of application, better residency and longer contact time onto the skin which confirm better absorption, and also to extend drug release. In-vitro drug release profile is an important parameter for the proper action for the anti-migraine formulation which necessitates an immediate drug release pattern followed by a slower rate. This was successfully established for the niosomal crosslinked GA-PVA patch as it showed a biphasic release pattern with a rapid initial release followed by a controlled rate up to 48 h (Fig. 3). The niosomal transdermal patch of midazolam also showed an initial burst release followed by a gradual sustained pattern [77].

The release kinetics of zolmitriptan from the ZOL transdermal patches (TPF_sp60/6−1:1_) followed Korsmeyer-Peppas model, as the magnitude of the regression coefficient “R^2^” is of highest value (0.993). On the other hand, the diffusion exponent “*n”* value was found to be less than 0.5 (0.316), suggesting quasi-Fickian diffusion of ZOL through the patch. Comparable results were reported with zolmitriptan buccal patch formulations with R^2^ ranging from 0.956 to 0.983 and “n” values 0.23–0.28 [78].

Our results confirmed stable ZOL lyophilized optimized niosomes (F_sp60/6−1:1_) formulation and ZOL transdermal patches (TPF_sp60/6−1:1_) after storage at 4 °C for 6 months. Hence it was exploited for in-vivo studies in rats, to compare the antimigraine action of the prepared patch formulation and oral ZOl solution and to investigate possible novel mechanisms for the antimigraine effect of ZOL.

In the present study, an experimental model was applied to mimic migraine headache pain; rats were administered NTG i.p., a NO donor, which induces an increased sensitivity to pain stimuli that reflects the increased sensitivity to painful stimuli in migraineurs. Its administration to rodents causes acute plantar mechanical hyperalgesia with progressive and sustained hyperalgesia [79]. It provokes a hyperalgesic state via second-order neurons in the nucleus trigeminal caudalis activation [80].

The intensity of migraine headache pain is one of the key items in identifying migraine severity [81]. CGRP and substance P are neurotransmitters play crucial roles in the development of pain and hyperalgesia in migraineurs and their levels were found to dramatically rise in patients suffering from migraine either with or without aura than in non-headache subjects [82]. Another neurotransmitter involved in pain transmission and hyperalgesia is NO. It is believed that variation in the blood flow of cerebral blood vessels caused by the vasodilatory effect of NO and CGRP is the main reason for migraine headache occurrence [83]. These neurotransmitters are released as a consequence of the activation of the trigeminovascular system and sensory transmission of nociceptive signals. Not only the neurotransmitters that are involved in trigeminovascular system activation, pro-inflammatory cytokines, such as TNF-α, are another important player in these processes. In addition, these cytokines play a role in stimulating meningeal nociceptors and thereby contribute to migraine pain [84]. C-fos is a validated marker to detect such neuronal activation that was determined in the present study [85]. In the current model for migraine induction, a significant elevation in the serum levels of substance P, NO, and TNF-α as well as brain levels of CGRP and c-fos was observed reflecting the success of the experimental model and induced hyperalgesia and matching earlier records [86]. These results were also verified by the behavioral tests results that showed a significant increase in pain symptoms including frequent cage climbing and head-scratching.

Treatment with a triptan, ZOL, significantly reduced the levels of pain neurotransmitters; CGRP, substance P, and NO, migraine neurogenic neuroinflammation marker; TNF-α that has a key role in migraine transition from episodic to chronic one [68], and trigeminovascular system activation marker; c-fos, compared to untreated rats with induced migraine. Consequently, pain symptoms assessed behaviorally were decreased which is in line with previous reports [87]. Triptans are 5-HT_1B/ID_ agonists that act on peripheral and/or central terminals of trigeminal ganglion neurons and inhibit pain neurotransmitters’ release to second-order neurons. This is considered one of the key mechanisms for pain relief by triptans as antimigraine drugs [11]. Niosomal ZOL formulation resulted in a further significant reduction of pain markers and symptoms reflecting enhanced bioavailability and probable better accessibility to the ZOL binding pocket of 5-HT_1B/ID_ located transmemberanous. It is reported that niosomes have good penetration to the cell membrane and high cellular uptake [88].

Photophobia is a common symptom of migraine and is considered one of the key criteria for migraine diagnosis according to the International Classification of Headache Disorders. Different visual *stimuli* are known to provoke migraine [89]. The main reason for photophobia is the existence of painful *stimuli* in certain eye areas including the conjunctiva, cornea, sclera, and uvea which are densely innervated with trigeminal fibers. These areas are exquisitely sensitive to pain that prominently involves the activity of the CGRP receptor [89]. In the present work, induction of migraine with NTG significantly increased light sensitivity and reduced time spent in light compared to negative control rats. This observation matches previous reports indicating that repeated (*n* = 5), but not single NTG injections, produced photophobia and decreased rats’ activity [49]. Treatment with either oral ZOL or ZOL transdermal patches (TPF_sp60/6−1:1_) significantly reduced NTG-induced photophobia, as predicted from ZOL influence on pain markers (especially CGRP) and pain symptoms, and these results are in accordance with previous studies. ZOL transdermal patches (TPF_sp60/6−1:1_) application resulted in a significant increase in time spent in light compared to the oral unformulated ZOL as a consequence of the more powerful ability to reduce pain neurotransmitters, especially CGRP which is the most important neurotransmitter involved in photophobia development [90].

Advances in molecular biology research highlighted the vital role of epigenetics in migraine pathogenesis, migraine chronification, and the therapeutic response of medications. Recently, epigenetics provided new insight into migraine pathogenesis and therapeutic response elucidation. Their mechanisms include methylation or histone acetylation to the cell genome under environmental signals’ influence and subsequent alteration to the functional output. Thus, environmental factors may directly trigger an acute migraine attack and may lead to epigenome alteration [4]. Key epigenetically altered genes in migraine include RAMP-1 and NPTX-2 genes [4]. RAMP-1 is rate-limiting for forming functional CGRP receptors and is mostly co-localized in trigeminal ganglion neurons. In a study performed by Seiler et al.., NTG infusion resulted in increased RAMP-1-immunopositive neurons reflecting an upregulation of functional CGRP receptors after NO donor pretreatment [91]. This is in agreement with the current results where migraine induction using NTG resulted in a 2.3-fold increase in RAMP-1 gene expression. Thus, CGRP selective antagonists that recognize RAMP-1 epitopes were developed and tested for migraine treatment as promising candidates to regulate the number of functional CGRP receptors. However, comparing the efficacy of such antagonists (e.g. telcagepant) to ZOL for migraine treatment revealed equipotency only at a high telcagepant dose (300 mg) [92]. This may indicate that migraine is best treated with medications with a multitude of mechanisms of action rather than using a high dose of a selective drug for a single mechanism. In our study, ZOL significantly reduced RAMP-1 mean gene expression in the brains of rats with NTG-induced migraine by 23% compared to untreated rats. The other tested gene was the NPTX-2 gene which plays an important role in synaptic plasticity regulation. Herein, ZOL oral treatment significantly reduced NPTX-2 mean gene expression by 37.6% relative to the untreated rats with NTG-induced migraine. To our knowledge, this is the first study that examined the influence of ZOL on RAMP-1 and NPTX-2 gene expression as an additional potential mechanism of action in migraineurs. The transdermal application of ZOL transdermal patches (TPF_sp60/6−1:1_) further significantly reduced RAMP-1 and NPTX-2 mean gene expression in rats’ brains by 31.3 and 50%, respectively, compared to the untreated rats with induced migraine, presumably due to enhanced brain delivery and accessibility to target molecules and transcription factors that may regulate these genes.

Another tested novel ZOL mechanism of action, beyond its well-known action on the 5-HT_1B/1D_ receptor, is the CB-1/MAPK pathway. CB-1 belongs to the endocannabinoid system that is widely distributed in the nervous system to regulate myriad physiological functions including pain and neuroinflammation. Growing evidence proposes the involvement of this system in the pathophysiology of migraine since CB-1 receptors contribute to the CGRP release from the trigeminal neurons [93]. In accordance with our results, it was also formerly proved that migraine induction experimentally using NTG significantly affects the endocannabinoid system through the CB-1 receptor [93], besides, NTG can significantly affect the MAPK signaling pathways [94]. NTG showed greater influence on MAPK expression relative to CB-1 receptor expression; i.e., the rise in CB-1/MAPK expressions is not parallel, probably because MAPK signaling cascades can be activated by many inflammatory mediators and by receptors other than CB-1 [95]. The ability of ZOL to modulate CB-1 was not tested before and in our study treatment with either oral ZOL or transdermal ZOL niosomes significantly decreased CB-1 expression in the brain tissue with no significant difference between them. The ability of ZOL to affect MAPK signaling was previously tested and was related to its agonistic activity on the 5-HT_1B/1D_ receptor with subsequent induction of specific phosphatases that are known negative regulators of MAPKs [95]. The more powerful ability of the transdermal ZOL niosomes to significantly decrease MAPK expression indicates more ability to induce such phosphatases.

One major aim of the study is to investigate the effect of the conventional oral ZOL and the ZOL transdermal patches (TPF_sp60/6−1:1_) on hemostatic pathways supposed to be altered in migraineurs. In the current investigation, platelet activation and coagulation markers were assessed peripherally and centrally. Peripherally in blood, a marker for platelet activation and production of secretory products; CD 41 [96], and two key clotting factors; thrombin (factor II) and factor X were assessed. Whereas in brain tissue, more platelet-related parameters were assessed including von Willebrand factor; an adhesive protein that contributes to platelet function by mediating thrombus formation initiation and progression at sites of vascular injury [97], and ADP; an important agonist for platelet aggregation that also induces platelet shape change and secretion from storage granules [98]. A vast amount of evidence suggests that migraineurs, particularly those with aura, have an increased risk for ischemic stroke due to the existence of hypercoagulable status in migraine. This hypercoagulable status is manifested by increased levels of von Willebrand factor antigen, fibrinogen, endothelial microparticles, and thrombin [99, 100]. In the current model of NTG-induced migraine in rats, all the tested platelet activation and coagulation markers were significantly increased confirming the existence of hypercoagulable status. A possible explanation of migraine-associated hypercoagulable status is migraine-induced oxidative stress and inflammation that results in endothelium dysfunction. Endothelial dysfunction results in increased vascular tone, thrombosis, and vascular permeability, and oxidative stress causes increased endothelin-1 activity that is not counterbalanced by NO. This oxidative stress may induce a hypercoagulable state that mainly disturbs platelet function [101]. Regarding the influence of ZOL on hemostatic mechanisms, data from the literature denoted no clear results; it was noted that ZOL causes no significant changes in platelet aggregation peripherally [102]. On the other hand, some reports referred to ZOL-induced ischemia that causes persistent cerebral perfusion deficits which may lead to platelet deposition and fibrin accumulation within the cerebral circulation [15]. ZOL significantly reduced the levels of all platelet aggregation and coagulation markers peripherally and centrally compared to untreated rats. However, the levels of all these markers are still significantly higher than the normal levels, except for serum CD41. Thus, ZOL is considered safe in low-risk migraineurs but should be avoided in those with vascular risk factors and prior cerebral or cardiac ischemias [103]. Since oxidative stress is considered the main cause for the hypercoagulable state that disturbs platelet function in migraineurs [101], the relief of such oxidative stress may be the mechanism by which ZOL has decreased the hypercoagulable state and coagulation markers levels. In accordance, ZOL was proven to alleviate oxidative stress in other neurological disorders including Alzheimer’s disease and Parkinsonism [104, 105]. The niosomal ZOL transdermal patches (TPF_sp60/6−1:1_) significantly further reduced the levels of all platelet aggregation and coagulation markers peripherally and centrally compared to the conventional oral ZOL solution. This may indicate the masking of ZOL vasoconstrictive action by its nanoformulation until delivery to the site of action. A previous study reported that niosomes their selves do not cause blood coagulation and more importantly do not generate oxygen-free radicles or induce oxidative stress whatever the electric charge of niosomes [37]. This means that ZOL niosomal formulation may be more safely administered to migraineurs with vascular risk factors and prior ischemia compared to the unformulated ZOL.

## Conclusion

Optimized ZOL niosomal formulation (F_sp60/6−1:1_) was successfully prepared and exhibited acceptable results: highest %EE (57.28%), acceptable PS (472.3 nm), and PDI (0.366). Then niosomal transdermal patch (TPF_sp60/6−1:1_) was prepared by solvent-casting technique by dispersing the lyophilized ZOL niosomal formulation (F_sp60/6−1:1_) into a PVA solution cross-linked with GA and followed by drying. ZOL niosomal transdermal patch (TPF_sp60/6−1:1_) was exploited for in-vivo studies and compared to ZOL solution. Novel mechanisms were proven to be exerted by ZOL in ameliorating migraine headache and pain such as decreasing the gene expression of key epigenetically altered genes in migraine; RAMP-1 and NPTX-2, and modulation of the endocannabinoid; CB-1/MAPK pathway. The transdermal delivery of niosomal ZOL (TPF_sp60/6−1:1_) showed significantly enhanced management of migraine pain symptoms and markers, decreased RAMP-1, NPTX-2, and CB-1/MAPK gene expression reflecting improved efficacy and brain receptor delivery. Additionally, this nanoformulation significantly opposed migraine-induced hypercoagulable status to a much greater extent than conventional ZOL solution has done.

For commercialization, clinical trials are recommended after fulfilling the required investigations, such as determination of the patch size, proper application site, probable side effects, dosing, frequency and duration of use. Challenges facing the production of nanoformulations including safety, cost, gaining FDA approval, must also be considered.

## Electronic supplementary material

Below is the link to the electronic supplementary material.

Supplementary Material 1

## Data Availability

The datasets generated during and/or analyzed during the current study are available from the corresponding author on reasonable request.

## References

[1] Puledda F, Silva EM, Suwanlaong K, Goadsby PJ. Migraine: from pathophysiology to treatment. J Neurol. 2023:1–13. 10.1007/s00415-023-11706-1 PMC10267278 37029836

[2] Stovner LJ, Nichols E, Steiner TJ, Abd-Allah F, Abdelalim A, Al-Raddadi RM, et al. Global, regional, and national burden of migraine and tension-type headache, 1990–2016: a systematic analysis for the global burden of Disease Study 2016. Lancet Neurol. 2018;17(11):954–76. 30353868 10.1016/S1474-4422(18)30322-3 PMC6191530

[3] Fan L, Wu Y, Wei J, Xia F, Cai Y, Zhang S, et al. Global, regional, and national time trends in incidence for migraine, from 1990 to 2019: an age-period-cohort analysis for the GBD 2019. J Headache Pain. 2023;24(1):79. 37391721 10.1186/s10194-023-01619-9 PMC10314517

[4] Zobdeh F, Eremenko II, Akan MA, Tarasov VV, Chubarev VN, Schiöth HB, et al. The epigenetics of Migraine. Int J Mol Sci. 2023;24(11):9127. 37298078 10.3390/ijms24119127 PMC10252316

[5] Mungoven TJ, Henderson LA, Meylakh N. Chronic migraine pathophysiology and treatment: a review of current perspectives. Front Pain Res. 2021;2:705276. 10.3389/fpain.2021.705276 PMC8915760 35295486

[6] Tajti J, Szok D, Csáti A, Szabó Á, Tanaka M, Vécsei L. Exploring novel therapeutic targets in the common pathogenic factors in migraine and neuropathic pain. Int J Mol Sci. 2023;24(4):4114. 36835524 10.3390/ijms24044114 PMC9959352

[7] Ibsen MS, Connor M, Glass M. Cannabinoid CB1 and CB2 receptor signaling and bias. Cannabis Cannabinoid Res. 2017;2(1):48–60. 28861504 10.1089/can.2016.0037 PMC5436336

[8] D’Addario C, Di Francesco A, Pucci M, Finazzi Agrò A, Maccarrone M. Epigenetic mechanisms and endocannabinoid signalling. FEBS J. 2013;280(9):1905–17. 23305292 10.1111/febs.12125

[9] Greco R, De Icco R, Demartini C, Zanaboni AM, Tumelero E, Sances G, et al. Plasma levels of CGRP and expression of specific microRNAs in blood cells of episodic and chronic migraine subjects: towards the identification of a panel of peripheral biomarkers of migraine? J Headache Pain. 2020;21:1–12. 33066724 10.1186/s10194-020-01189-0 PMC7565351

[10] Ahmed Kassem A. Formulation approaches of triptans for management of migraine. Curr Drug Deliv. 2016;13(6):882–98. 27109335 10.2174/1567201813666160425112600

[11] MacLennan SJ, Cambridge D, Whiting MV, Marston C, Martin GR. Cranial vascular effects of zolmitriptan, a centrally active 5-HT1B/1D receptor partial agonist for the acute treatment of migraine. Eur J Pharmacol. 1998;361(2–3):191–7. 9865508 10.1016/s0014-2999(98)00727-4

[12] Hassan DH, Shohdy JN, El-Setouhy DA, El-Nabarawi M, Naguib MJ. Compritol-based nanostrucutured lipid carriers (NLCs) for augmentation of zolmitriptan bioavailability via the transdermal route: in vitro optimization, ex vivo permeation, in vivo pharmacokinetic study. Pharmaceutics. 2022;14(7):1484. 35890379 10.3390/pharmaceutics14071484 PMC9315618

[13] Tawfik MA, Eltaweel MM, Fatouh AM, Shamsel-Din HA, Ibrahim AB. Brain targeting of zolmitriptan via transdermal terpesomes: statistical optimization and in vivo biodistribution study by 99mTc radiolabeling technique. Drug Delivery Translational Res. 2023;13(12):3059–76. 10.1007/s13346-023-01373-0 PMC10624728 37273147

[14] Girotra P, Singh SK, Kumar G. Development of zolmitriptan loaded PLGA/poloxamer nanoparticles for migraine using quality by design approach. Int J Biol Macromol. 2016;85:92–101. 26724690 10.1016/j.ijbiomac.2015.12.069

[15] Nicolas SND, Triptans. In: StatPearls [Internet]. Treasure Island (FL); 2024.

[16] Adhami F, Liao G, Morozov YM, Schloemer A, Schmithorst VJ, Lorenz JN, et al. Cerebral ischemia-hypoxia induces intravascular coagulation and autophagy. Am J Pathol. 2006;169(2):566–83. 16877357 10.2353/ajpath.2006.051066 PMC1780162

[17] Liu C, Fang L. Drug in adhesive patch of zolmitriptan: formulation and in vitro/in vivo correlation. AAPS PharmSciTech. 2015;16:1245–53. 25771739 10.1208/s12249-015-0303-3 PMC4666264

[18] Gaikwad SS, Zanje AL, Somwanshi JD. Advancements in Transdermal Drug Delivery: a comprehensive review of physical penetration enhancement techniques. Int J Pharm. 2024:123856. 10.1016/j.ijpharm.2024.123856 38281692

[19] Guy RH. Drug delivery to and through the skin. Drug Delivery Translational Res. 2024:1–9. 10.1007/s13346-024-01614-w PMC11208237 38837116

[20] Ramadon D, McCrudden MT, Courtenay AJ, Donnelly RF. Enhancement strategies for transdermal drug delivery systems: current trends and applications. Drug Delivery Translational Res. 2021:1–34. 10.1007/s13346-021-00909-6 PMC7817074 33474709

[21] Ita K. Microneedles. In: Ita K, editor. Transdermal Drug Delivery: concepts and application. Academic; 2020. pp. 143–81.

[22] Ita K, Ukaoma M. Progress in the transdermal delivery of antimigraine drugs. J Drug Deliv Sci Technol. 2022;68:103064.

[23] Zhou X, Hao Y, Yuan L, Pradhan S, Shrestha K, Pradhan O, et al. Nano-formulations for transdermal drug delivery: a review. Chin Chem Lett. 2018;29(12):1713–24.

[24] Xu P, Huang S, Zhang H, Mao C, Zhou XE, Cheng X, et al. Structural insights into the lipid and ligand regulation of serotonin receptors. Nature. 2021;592(7854):469–73. 33762731 10.1038/s41586-021-03376-8

[25] Hami Z. A brief review on advantages of nano-based drug delivery systems. Annals Military Health Sci Res. 2021;19(1).

[26] Abd El-Halim SM, Mamdouh MA, Eid SM, Ibrahim BM, Aly Labib DA, Soliman SM. The potential synergistic activity of zolmitriptan combined in new self-nanoemulsifying drug delivery systems: atr-ftir real-time fast dissolution monitoring and pharmacodynamic assessment. Int J Nanomed. 2021:6395–412. 10.2147/IJN.S325697 PMC8456549 34566412

[27] Shafi H, Rashid R, Rather S-u, Reddy DS, Azmi L, Abdal-hay A, et al. Super disintegrating oromucosal nanofiber patch of zolmitriptan for rapid delivery and efficient brain targeting. Chem Eng J. 2023;463:142481.

[28] Mandlik SK, Ranpise NS, Mohanty BS, Chaudhari PR. A coupled bimodal SPECT-CT imaging and brain kinetics studies of zolmitriptan-encapsulated nanostructured polymeric carriers. Drug Delivery Translational Res. 2018;8:797–805. 10.1007/s13346-017-0474-4 29380155

[29] Mostafa DAE, Khalifa MK, Gad SS. Zolmitriptan Brain targeting via intranasal route using solid lipid nanoparticles for migraine therapy: Formulation, characterization, in-vitro and In-vivo Assessment. Int J App Pharm. 2020;12(2):86–93.

[30] Jha S, Mishra D. Evaluation of Brain Targeting potential of Zolmitriptan Mucoadhesive nanoparticles for Intranasal Drug Delivery. Pharm Nanatechnol. 2022;10(2):113–24. 10.2174/2211738510666220303160414 35240970

[31] Abdou EM, Kandil SM, El Miniawy HM. Brain targeting efficiency of antimigrain drug loaded mucoadhesive intranasal nanoemulsion. Int J Pharm. 2017;529(1–2):667–77. 28729175 10.1016/j.ijpharm.2017.07.030

[32] Tawfik MA, Eltaweel MM, Farag MM, Shamsel-Din HA, Ibrahim AB. Sonophoresis-assisted transdermal delivery of antimigraine-loaded nanolipomers: Radio-tracking, histopathological assessment and in-vivo biodistribution study. Int J Pharm. 2023;644:123338. 37607646 10.1016/j.ijpharm.2023.123338

[33] Mohamed MI, Abdelbary AA, Kandil SM, Mahmoud TM. Preparation and evaluation of optimized zolmitriptan niosomal emulgel. Drug Dev Ind Pharm. 2019;45(7):1157–67. 30919700 10.1080/03639045.2019.1601737

[34] Farmoudeh A, Akbari J, Saeedi M, Ghasemi M, Asemi N, Nokhodchi A. Methylene blue-loaded niosome: preparation, physicochemical characterization, and in vivo wound healing assessment. Drug Delivery Translational Res. 2020;10:1428–41. 10.1007/s13346-020-00715-6 PMC7447683 32100265

[35] Yasamineh S, Yasamineh P, Kalajahi HG, Gholizadeh O, Yekanipour Z, Afkhami H, et al. A state-of-the-art review on the recent advances of niosomes as a targeted drug delivery system. Int J Pharm. 2022;624:121878. 35636629 10.1016/j.ijpharm.2022.121878

[36] Muzzalupo R, Tavano L. Niosomal drug delivery for transdermal targeting: recent advances. Research and reports in transdermal drug delivery. 2015:23–33.

[37] Moser P, Marchand-Arvier M, Labrude P, Vigneron C. Hemoglobin niosomes. II. In vitro interactions of plasma proteins and phagocytes. Pharm Acta Helv. 1990;65(3):82–92. 2185479

[38] Shilakari Asthana G, Sharma PK, Asthana A. In vitro and in vivo evaluation of niosomal formulation for controlled delivery of clarithromycin. Scientifica. 2016;2016. 10.1155/2016/6492953 PMC4884864 27293976

[39] Hassan A. Effective surfactants blend concentration determination for o/w emulsion stabilization by two nonionic surfactants by simple linear regression. Indian J Pharm Sci. 2015;77(4):461. 26664063 10.4103/0250-474x.164773 PMC4649784

[40] Abou Youssef NAH, Kassem AA, Farid RM, Ismail FA, Magda Abd Elsamea E-M, Boraie NA. A novel nasal almotriptan loaded solid lipid nanoparticles in mucoadhesive in situ gel formulation for brain targeting: Preparation, characterization and in vivo evaluation. Int J Pharm. 2018;548(1):609–24. 30033394 10.1016/j.ijpharm.2018.07.014

[41] Makled S, Nafee N, Boraie N. Nebulized solid lipid nanoparticles for the potential treatment of pulmonary hypertension via targeted delivery of phosphodiesterase-5-inhibitor. Int J Pharm. 2017;517(1–2):312–21. 27979766 10.1016/j.ijpharm.2016.12.026

[42] Figueiredo KC, Alves TL, Borges CP. Poly (vinyl alcohol) films crosslinked by glutaraldehyde under mild conditions. J Appl Polym Sci. 2009;111(6):3074–80.

[43] Mishra V, Mahor S, Rawat A, Dubey P, Gupta PN, Singh P, et al. Development of novel fusogenic vesosomes for transcutaneous immunization. Vaccine. 2006;24(27–28):5559–70. 16730102 10.1016/j.vaccine.2006.04.030

[44] Kriplani P, Guarve K, Baghel US. Formulation optimization and characterization of transdermal film of curcumin by response surface methodology. Chin Herb Med. 2021;13(2):274–85. 36117499 10.1016/j.chmed.2020.12.001 PMC9476792

[45] Ananda PWR, Elim D, Zaman HS, Muslimin W, Tunggeng MGR, Permana AD. Combination of transdermal patches and solid microneedles for improved transdermal delivery of primaquine. Int J Pharm. 2021;609:121204. 34662646 10.1016/j.ijpharm.2021.121204

[46] Fetih G, Ibrahim M, Amin M. Design and characterization of transdermal films containing ketorolac tromethamine. Int J PharmTech Res. 2011;3(1):449–58.

[47] Edition E. Guide for the care and use of laboratory animals. Washington: the national academies; 2011.

[48] Sun S, Zheng G, Zhou D, Zhu L, He X, Zhang C, et al. Emodin interferes with nitroglycerin-induced migraine in rats through cgmp-pkg pathway. Front Pharmacol. 2021;12:758026. 34744735 10.3389/fphar.2021.758026 PMC8563583

[49] Sufka KJ, Staszko SM, Johnson AP, Davis ME, Davis RE, Smitherman TA. Clinically relevant behavioral endpoints in a recurrent nitroglycerin migraine model in rats. J Headache Pain. 2016;17(1):1–7. 10.1186/s10194-016-0624-y PMC4837195 27093871

[50] Thrombin activity assay kit. https://www.abcam.com/en-us/products/assay-kits/thrombin-activity-assay-ab234620).

[51] (ADP Assay kit (Colorimetric/Fluorometric). https://www.abcam.com/en-us/products/assay-kits/adp-assay-kit-colorimetric-fluorometric-ab83359

[52] Nitric Oxide Assay kit (Colorimetric). https://www.abcam.com/en-us/products/assay-kits/nitric-oxide-assay-kit-colorimetric-ab65328

[53] Rat Proto-oncogene c-Fos ELISA Kit. https://www.assaygenie.com/content/ELISA%20Genie/EB/RTEB0846.pdf

[54] Rat calcitonin gene related peptide,CGRP ELISA Kit. https://www.cusabio.com/uploadfile/Ins/CSB-E08211r.pdf

[55] Rat ITGA2B / CD41 ELISA Kit. https://www.assaygenie.com/rat-itga2b-cd41-elisa-kit/

[56] Rat VWF, (Von Willebrand Factor) ELISA Kit. https://file.elabscience.com/Manual/elisa_kits/E-EL-R1079-Elabscience.pdf

[57] El-Mezayen NS, Attia MAM, Shafik MY, Gowied HG, Abdel‐Aal HA, Abdel‐Hady SM, et al. Reactive astrocytes targeting with oral vitamin A: efficient neuronal regeneration for Parkinson’s disease treatment and reversal of associated liver fibrosis. CNS Neurosci Ther. 2023;29(8):2111–28. 36949616 10.1111/cns.14179 PMC10352881

[58] Mabrouk AA, El-Mezayen NS, Awaad AK, Tadros MI, El-Gazayerly ON, El-Refaie WM. Novel celecoxib-loaded chitosan-fucoidan nanoparticles as potential immunotherapy for oral squamous cell carcinoma: mechanistic insights. J Drug Deliv Sci Technol. 2023;81:104228.

[59] Rat Substance P, SP ELISA Kit. https://www.cusabio.com/ELISA-Kit/Rat-Substance-PSP-ELISA-Kit-105489.html

[60] Rat, TNF-α ELISA kit. https://www.cusabio.com/ELISA-Kit/Rat-TNF-%CE%B1-ELISA-kit-109331.html

[61] Rat coagulation factor,F ELISA Kit. https://www.cusabio.com/ELISA-Kit/Rat-coagulation-factor-%E2%85%A9F%E2%85%A9-ELISA-Kit-77156.html

[62] Ibrahim RS, El-Mezayen NS, El-Banna AA. Alleviation of liver cirrhosis and associated portal-hypertension by Astragalus species in relation to their UPLC-MS/MS metabolic profiles: a mechanistic study. Sci Rep. 2022;12(1):11884. 35831335 10.1038/s41598-022-15958-1 PMC9279505

[63] Moustafa MA, El-Refaie WM, Elnaggar YS, El-Mezayen NS, Awaad AK, Abdallah OY. Fucoidan/hyaluronic acid cross-linked zein nanoparticles loaded with fisetin as a novel targeted nanotherapy for oral cancer. Int J Biol Macromol. 2023;241:124528. 37086764 10.1016/j.ijbiomac.2023.124528

[64] Ibrahim RS, El-Mezayen NS, Khairy A, Zaatout HH, Hammoda HM, Metwally AM. Biologically-guided isolation of natural lead antithyroid drug from Medicago sativa L. sprouts and its toxic profile in comparison with propylthiouracil. J Food Drug Anal. 2020;28(3):407. 35696097 10.38212/2224-6614.1242 PMC9261795

[65] Neeb L, Reuter U. Nitric oxide in migraine. CNS Neurol Disorders-Drug Targets (Formerly Curr Drug Targets-CNS Neurol Disorders). 2007;6(4):258–64. 10.2174/187152707781387233 17691982

[66] Mitsikostas DD, del Rio MS. Receptor systems mediating c-fos expression within trigeminal nucleus caudalis in animal models of migraine. Brain Res Rev. 2001;35(1):20–35. 11245884 10.1016/s0165-0173(00)00048-5

[67] Lassen L, Haderslev P, Jacobsen V, Iversen HK, Sperling B, Olesen J. CGRP may play a causative role in migraine. Cephalalgia. 2002;22(1):54–61. 11993614 10.1046/j.1468-2982.2002.00310.x

[68] Sudershan A, Sudershan S, Sharma I, Kumar H, Panjaliya RK, Kumar P. Role of TNF-α in the Pathogenesis of Migraine. Pain Research and Management. 2024;2024. 10.1155/2024/1377143 PMC10781531 38213956

[69] Shah P, Goodyear B, Haq A, Puri V, Michniak-Kohn B. Evaluations of quality by design (QbD) elements impact for developing niosomes as a promising topical drug delivery platform. Pharmaceutics. 2020;12(3):246. 32182792 10.3390/pharmaceutics12030246 PMC7150869

[70] Kumar GP, Rajeshwarrao P. Nonionic surfactant vesicular systems for effective drug delivery—an overview. Acta Pharm Sinica B. 2011;1(4):208–19.

[71] Uchegbu IF, Vyas SP. Non-ionic surfactant based vesicles (niosomes) in drug delivery. Int J Pharm. 1998;172(1–2):33–70.

[72] Shahiwala A, Misra A. Studies in topical application of niosomally entrapped nimesulide. J Pharm Pharm Sci. 2002;5(3):220–5. 12553889

[73] Ruckmani K, Sankar V. Formulation and optimization of zidovudine niosomes. AAPS PharmSciTech. 2010;11:1119–27. 20635228 10.1208/s12249-010-9480-2 PMC2974162

[74] El-Mahdy M, Mohamed E-EM, Saddik MS, Ali MF, El-Sayed AM. Formulation and clinical evaluation of niosomal methylene blue for successful treatment of acne. J Adv Biomedical Pharm Sci. 2020;3(3):116–26.

[75] SP ST, Mothilal M, Damodharan N, Jaison D. Screening and optimization of valacyclovir niosomes by design of experiments. Int J App Pharm 2018:79–85.

[76] Alshawwa SZ, Labib GS, Badr-Eldin SM, Kassem AA. Solid lipid Lyo-Nanosuspension: a promising stabilized oral delivery system for the antihyperglycemic extract of mistletoe Plicosepalus acacia. Saudi Pharm J. 2023;31(8):101689. 37457370 10.1016/j.jsps.2023.06.022 PMC10339052

[77] Shefrin S, Sreelaxmi C, Vishnu V, Sreeja C. Antiepileptic drug loaded niosomal transdermal patch for enhanced skin permeation. Int J App Pharm. 2019;11(2):31–43.

[78] Shiledar RR, Tagalpallewar AA, Kokare CR. Formulation and in vitro evaluation of xanthan gum-based bilayered mucoadhesive buccal patches of zolmitriptan. Carbohydr Polym. 2014;101:1234–42. 24299896 10.1016/j.carbpol.2013.10.072

[79] Greco R, Demartini C, Zanaboni AM, Tassorelli C. Chronic and intermittent administration of systemic nitroglycerin in the rat induces an increase in the gene expression of CGRP in central areas: potential contribution to pain processing. J Headache Pain. 2018;19:1–8. 30003352 10.1186/s10194-018-0879-6 PMC6043463

[80] Greco R, Tassorelli C, Sandrini G, Di Bella P, Buscone S, Nappi G. Role of calcitonin gene-related peptide and substance P in different models of pain. Cephalalgia. 2008;28(2):114–26. 18197882 10.1111/j.1468-2982.2007.01468.x

[81] El Hasnaoui A, Vray M, Richard A, Nachit-Ouinekh F, Boureau F, Group M. Assessing the severity of migraine: development of the MIGSEV scale. Headache: J Head Face Pain. 2003;43(6):628–35. 10.1046/j.1526-4610.2003.03105.x 12786922

[82] Fusayasu E, Kowa H, Takeshima T, Nakaso K, Nakashima K. Increased plasma substance P and CGRP levels, and high ACE activity in migraineurs during headache-free periods. Pain. 2007;128(3):209–14. 17123735 10.1016/j.pain.2006.09.017

[83] Aggarwal M, Puri V, Puri S. Serotonin and CGRP in migraine. Annals Neurosciences. 2012;19(2):88–94. 10.5214/ans.0972.7531.12190210 PMC4117050 25205974

[84] Thuraiaiyah J, Erritzøe-Jervild M, Al-Khazali HM, Schytz HW, Younis S. The role of cytokines in migraine: a systematic review. Cephalalgia. 2022;42(14):1565–88. 35962530 10.1177/03331024221118924

[85] Wu S, Ren X, Zhu C, Wang W, Zhang K, Li Z, et al. A c-Fos activation map in nitroglycerin/levcromakalim-induced models of migraine. J Headache Pain. 2022;23(1):128. 36180824 10.1186/s10194-022-01496-8 PMC9524028

[86] Park S, Jung H, Han S-W, Lee S-H, Sohn J-H. Differences in Neuropathology between Nitroglycerin-Induced mouse models of episodic and chronic migraine. Int J Mol Sci. 2024;25(7):3706. 38612517 10.3390/ijms25073706 PMC11011425

[87] Thomaides T, Karagounakis D, Spantideas A, Katelanis S. Transcranial doppler in migraine attacks before and after treatment with oral zolmitriptan or sumatriptan. Headache: J Head Face Pain. 2003;43(1):54–8. 10.1046/j.1526-4610.2003.03009.x 12864759

[88] Kauslya A, Borawake PD, Shinde JV, Chavan RS. Niosomes: a novel carrier drug delivery system. J Drug Delivery Ther. 2021;11(1):162–70.

[89] Digre KB, Brennan K. Shedding light on photophobia. J Neuroophthalmol. 2012;32(1):68–81. 22330853 10.1097/WNO.0b013e3182474548 PMC3485070

[90] Mason BN, Kaiser EA, Kuburas A, Loomis M-CM, Latham JA, Garcia-Martinez LF, et al. Induction of migraine-like photophobic behavior in mice by both peripheral and central CGRP mechanisms. J Neurosci. 2017;37(1):204–16. 28053042 10.1523/JNEUROSCI.2967-16.2016 PMC5214631

[91] Seiler K, Nusser JI, Lennerz JK, Neuhuber WL, Messlinger K. Changes in calcitonin gene-related peptide (CGRP) receptor component and nitric oxide receptor (sGC) immunoreactivity in rat trigeminal ganglion following glyceroltrinitrate pretreatment. J Headache Pain. 2013;14:1–14. 24004534 10.1186/1129-2377-14-74 PMC3847895

[92] Moore EL, Salvatore CA. Targeting a family B GPCR/RAMP receptor complex: CGRP receptor antagonists and migraine. Br J Pharmacol. 2012;166(1):66–78. 21871019 10.1111/j.1476-5381.2011.01633.x PMC3415638

[93] Kilinc E, Ankarali S, Torun IE, Dagistan Y. Receptor mechanisms mediating the anti-neuroinflammatory effects of endocannabinoid system modulation in a rat model of migraine. Eur J Neurosci. 2022;55(4):1015–31. 32639078 10.1111/ejn.14897

[94] Mao Q, Cui Y, Du H, Wu J, Zhou M, Ouyang H, et al. San Pian decoction can treat nitroglycerin-induced migraine in rats by inhibiting the PI3K/AKT and MAPK signaling pathways. J Ethnopharmacol. 2022;296:115470. 35738471 10.1016/j.jep.2022.115470

[95] Durham PL, Russo AF. New insights into the molecular actions of serotonergic antimigraine drugs. Pharmacol Ther. 2002;94(1–2):77–92. 12191595 10.1016/s0163-7258(02)00173-0

[96] Ghoshal K, Bhattacharyya M. Overview of platelet physiology: its hemostatic and nonhemostatic role in disease pathogenesis. Sci World J. 2014;2014. 10.1155/2014/781857 PMC3960550 24729754

[97] Ruggeri ZM. Von Willebrand factor. Curr Opin Hematol. 2003;10(2):142–9. 12579041 10.1097/00062752-200303000-00008

[98] Puri RN, Colman RW, Liberman MA. ADP-lnduced platelet activation. Crit Rev Biochem Mol Biol. 1997;32(6):437–502. 9444477 10.3109/10409239709082000

[99] Tietjen GE, Collins SA. Hypercoagulability and migraine. Headache: J Head Face Pain. 2018;58(1):173–83. 10.1111/head.13044 28181217

[100] Hering-Hanit R, Friedman Z, Schlesinger I, Ellis M. Evidence for activation of the coagulation system in migraine with aura. Cephalalgia. 2001;21(2):137–9. 11422096 10.1046/j.1468-2982.2001.00181.x

[101] Paolucci M, Altamura C, Vernieri F. The role of endothelial dysfunction in the pathophysiology and cerebrovascular effects of migraine: a narrative review. J Clin Neurol (Seoul Korea). 2021;17(2):164. 10.3988/jcn.2021.17.2.164 PMC8053543 33835736

[102] Evers S, Heuel T, Frese A, Akova-Öztürk E, Husstedt I. The impact of different antimigraine compounds on platelet and erythrocyte aggregation. Cephalalgia. 2006;26(8):920–4. 16886927 10.1111/j.1468-2982.2006.01146.x

[103] Tietjen GE. The risk of stroke in patients with migraine and implications for migraine management. CNS Drugs. 2005;19:683–92. 16097850 10.2165/00023210-200519080-00004

[104] Tripathi AS, Fatima N, Tripathi P, Tripathi R, Alka, Zaki ME, et al. Beneficial effect of 5-HT1b/1d agonist on Parkinson’s disease by modulating glutamate and reducing deposition of α‐synuclein. J Biochem Mol Toxicol. 2024;38(1):e23627. 38229316 10.1002/jbt.23627

[105] Tripathi AS, Bansod P, Swathi K. Activation of 5-HT 1b/d receptor restores the cognitive function by reducing glutamate release, deposition of β-amyloid and TLR-4 pathway in the brain of scopolamine-induced dementia in rat. J Pharm Pharmacol. 2021;73(12):1592–8. 34244776 10.1093/jpp/rgab095

