# Supplementary figures and images for: Zolmitriptan niosomal transdermal patches: combating migraine via epigenetic and endocannabinoid pathways and reversal of migraine hypercoagulability
Source: Drug Deliv Transl Res. 2024 Nov 5;15(6):2179–99. doi: 10.1007/s13346-024-01731-6 (PMC12037682; doi:10.1007/s13346-024-01731-6)

**Supplementary file**


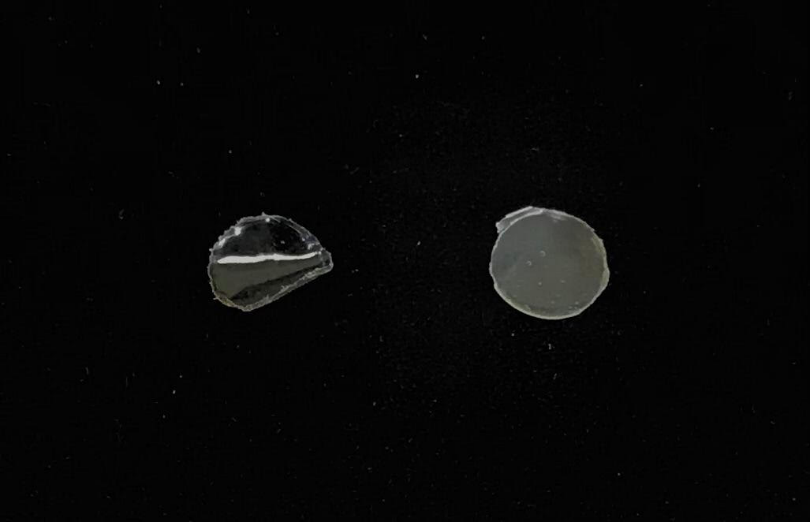


**A**

**B**

Photographs of:

1. *Placebo* patch
2. Zolmitriptan patch

Supplement: Supplementary file 1 — Supplementary Material 1 [file 13346_2024_1731_MOESM1_ESM.docx]
